# *Faecalibaculum rodentium* Alleviates Ionizing Radiation‐Induced Damage in Mice by Improving Intestinal Integrity and Hematopoiesis via Its Metabolite Butyrate

**DOI:** 10.1002/advs.202509383

**Published:** 2025-10-13

**Authors:** Hanyong Zhu, Hui Guo, Na Sun, Rui Xiao, Binbin Ji, Ruihan Jiang, Fuxing Dong, Chen Yao, Xuerong Wang, Rongqing Li, Jie Zhao, Xueqin Li, Shengzhe Gong, Yongqin Qin, Xin Chen, Yuchen Pan, Takayuki Ikezoe, Jing Yang

**Affiliations:** ^1^ Jiangsu International Laboratory of Immunity and Metabolism Jiangsu Province Key Laboratory of Immunity and Metabolism The Department of Pathogenic Biology and Immunology Basic Medicine School, Xuzhou Medical University Xuzhou Jiangsu 221004 China; ^2^ Department of Laboratory Medicine Hospital of Southwest Petroleum University Chengdu Sichuan 610500 China; ^3^ National Experimental Demonstration Center for Basic Medicine Education Xuzhou Medical University Xuzhou Jiangsu 221004 China; ^4^ Department of Clinical Laboratory The Affiliated Jinhua Hospital Zhejiang University School of Medicine Jinhua Zhejiang 321000 China; ^5^ Public Experimental Research Center Xuzhou Medical University Xuzhou Jiangsu 221004 China; ^6^ Fujian Key Laboratory of Laboratory Medicine the First Affiliated Hospital of Fujian Medical University the School of Medical Technology and Engineering Fujian Medical University Fuzhou 350122 China; ^7^ Department of Hematology Fukushima Medical University Fukushima 960‐1295 Japan

**Keywords:** apoptosis, butyrate, F. rodentium, gut hemostasis, HSC

## Abstract

The gut microbiota is key to mitigating ionizing radiation (IR)–induced injuries; however, the specific species involved in and the molecular mechanisms remain elusive. Mitochondrial dynamics affect gut microbiota diversity. To identify the specific species involved in the radioprotective effect, we performed mitochondrial proteomic profiling of mouse intestinal epithelial cells and identified the accumulation of signal transducer and activator of transcription 3 (STAT3). Using mitochondrial STAT3 knock‐in mice, we observed the abundance of the probiotic *Faecalibaculum rodentium* and its metabolite butyrate decreased in parallel with increased sensitivity to IR. Supplementation with *Faecalibaculum rodentium* or butyrate attenuated IR‐induced intestinal barrier dysfunction, enhanced hematopoietic recovery, and prolonged survival. Butyrate is found to exert dual protective effects: It increases tight junction proteins, such as zonula occludens‐1 (ZO‐1) and occludin, and the defense factor levels to reinforce intestinal integrity. Furthermore, it sustains extracellular regulated protein kinases (ERK)‐mediated pyruvate kinase isozyme type M2 (PKM2) nuclear localization, thereby attenuating p53‐dependent apoptotic signaling in hematopoietic stem cells and ultimately prolonging mouse survival. These findings indicate that *Faecalibaculum rodentium*‐derived butyrate confers radioprotection by maintaining the intestinal barrier and hematopoietic regeneration, suggesting a promising microbiota‐directed therapeutic strategy against radiation‐induced injury.

## Graphical abstract

The model shows the probiotics screening and underlying mechanisms of *F. rodentiumn* in alleviating IR‐induced tissue injury. Stress‐induced STAT3 mitochondria localization decreases *F. rodentium* abundance and butyrate concentration. Supplementation of *F. rodentium* or butyrate increases tight junction protein and defensive factors, blocks ERK/PKM2/p53 signaling‐mediated apoptosis of hematopoietic progenitors, reinforcing intestinal integrity and promoting hematopoietic recovery, which ultimately prolongs mice survival.

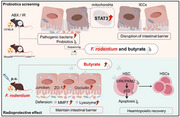

## 1 Introduction

The gut microbiota greatly influences host physiology in both homeostasis and pathological states via microbial components, microbiota‐derived metabolites, and nutrient regulation.^[^ ^1^, ^2^, ^3^, ^4^ ^]^ Emerging evidence indicates that the gut microbiome plays a critical role in modulating hematopoietic recovery and that it is a therapeutic target for mitigating ionizing radiation (IR)‐induced tissue injury.

Myeloid cell populations have been found to be positively correlated with gut microbial diversity.^[^ ^1^ ^]^ Under steady‐state conditions, microbiota‐derived molecules enhance inflammatory cytokine production by C‐X3‐C motif chemokine receptor 1 (CX3CR1)‐positive mononuclear cells, thereby regulating basal progenitor expansion.^[^ ^2^ ^]^ Furthermore, the gut microbiome is indispensable for maintaining neutrophil homeostasis and regulating aging, with intricate interactions occurring between the gut microbiota and hematopoietic cells that contribute to inflammatory disorders and malignancies.^[^ ^3^, ^4^, ^5^, ^6^, ^7^ ^]^ Broad‐spectrum antibiotic (ABX) administration compromises hematopoietic progenitor maintenance and granulocyte maturation.^[^ ^8^ ^]^ Recent research has shown that, under stress conditions, the gut microbiome and its metabolite butyrate augment the bone marrow (BM) macrophage capacity for recycling and provision of iron to hematopoietic stem cells (HSCs), thereby influencing HSC stemness.^[^ ^9^ ^]^

The gastrointestinal (GI) tract and BM show the greatest sensitivity to IR. IR‐induced injuries to these systems are the main causes of IR‐related mortality. During IR exposure, Lachnospiraceae family strains and their metabolites (e.g., propionate and butyrate) are crucial for promoting GI repair and accelerating hematopoietic recovery.^[^ ^10^ ^]^ Furthermore, nicotinamide riboside, a vitamin B3 derivative and microbiota metabolite, preserves HSC function ^[^ ^11^ ^]^ and alleviates IR‐induced hematopoietic injury,^[^ ^12^ ^]^ underscoring the pivotal regulatory roles of the microbiota and its metabolites in radioprotection. However, the species critical for mediating radioprotection remain unclear.

The GI tract hosts a complex microbial ecosystem comprising an estimated 3 million microbial genes,^[^ ^13^, ^14^ ^]^ which poses significant challenges in identifying specific bacterial species essential for radioprotection without the use of appropriate experimental models. In addition to dietary patterns, intestinal epithelial cell (IEC)‐mediated metabolic processes and mucosal defense profoundly influence gut microbial composition.^[^ ^15^ ^]^ Given the central role of the mitochondria in IECs,^[^ ^16^ ^]^ cellular stress responses that induce mitochondrial alterations, including mitochondrial protein relocalization, could modulate microbiota dynamics.^[^ ^17^, ^18^ ^]^ Thus, these previous studies suggest the feasibility of screening the mitochondrial proteomic profiles of IECs for developing targeted murine models in order to identify the microbiota essential for radioprotection.

In this study, to screen the key microbiota strain(s) essential for radioprotection, we performed mitochondrial proteomic profiling of IECs and identified signal transducer and activator of transcription 3 (STAT3) localized in IEC mitochondria. Furthermore, we used inducible mitochondrial STAT3 knock‐in mice to identify key microbial species involved in radioprotection and to elucidate their mechanistic roles in this process.

## 2 Results

### 2.1 Mitochondrial STAT3 Knock‐In Mouse Model was Established for Selecting Potential Probiotics

We first confirmed that the gut flora is essential for mediating hematopoiesis and IR sensitivity. The mice were subjected to ABX treatment, and the α‐diversity, β‐diversity, and the relative abundances of the probiotic family were compared to those in control mice, both with and without IR exposure (Figure S1A–F, Supporting Information). ABX treatment disrupted crypt‐villus architecture and decreased the hematopoietic cell count (Figure S1G,H, Supporting Information), and exacerbated IR‐induced mortality, with concomitant crypt‐villus loss, hematopoietic cell reduction, and diminished HSC colony formation (Figure S1I–K, Supporting Information); these findings verified the importance of the gut microbiota in alleviating IR‐induced damage.

Mitochondrial alterations in the intestinal epithelium can promote gut microbiota dysbiosis,^[^ ^19^, ^20^ ^]^ affecting both the GI and hematopoietic systems.^[^ ^8^ ^]^ Using mass spectrometry analysis, we sought to identify the critical proteins controlling mitochondrial alterations in order to establish a mouse model for species screening. STAT3, but not STAT1/2, accumulated in IEC mitochondria after ABX treatment and IR exposure (Table S1 and Figure S1L,M, Supporting Information), indicating that STAT3 mitochondrial localization could affect the gut flora.

To investigate the relationship between mitochondrial STAT3 and the gut microbiota, we utilized whole‐body mitochondrial STAT3 knock‐in mice.^[^ ^21^ ^]^ Compared with WT mice, our findings showed mitochondrial STAT3 knock‐in altered gut microbial diversity (α/β‐diversity) and probiotic reduction (Figure S2A–E, Supporting Information). Moreover, these mice exhibited 100% mortality within 14 days after IR, presenting with BM aplasia and GI syndrome^[^ ^19^ ^]^ characterized by hematopoietic element depletion and crypt‐villus atrophy (Figure S2F,G, Supporting Information).

**Figure 1:**
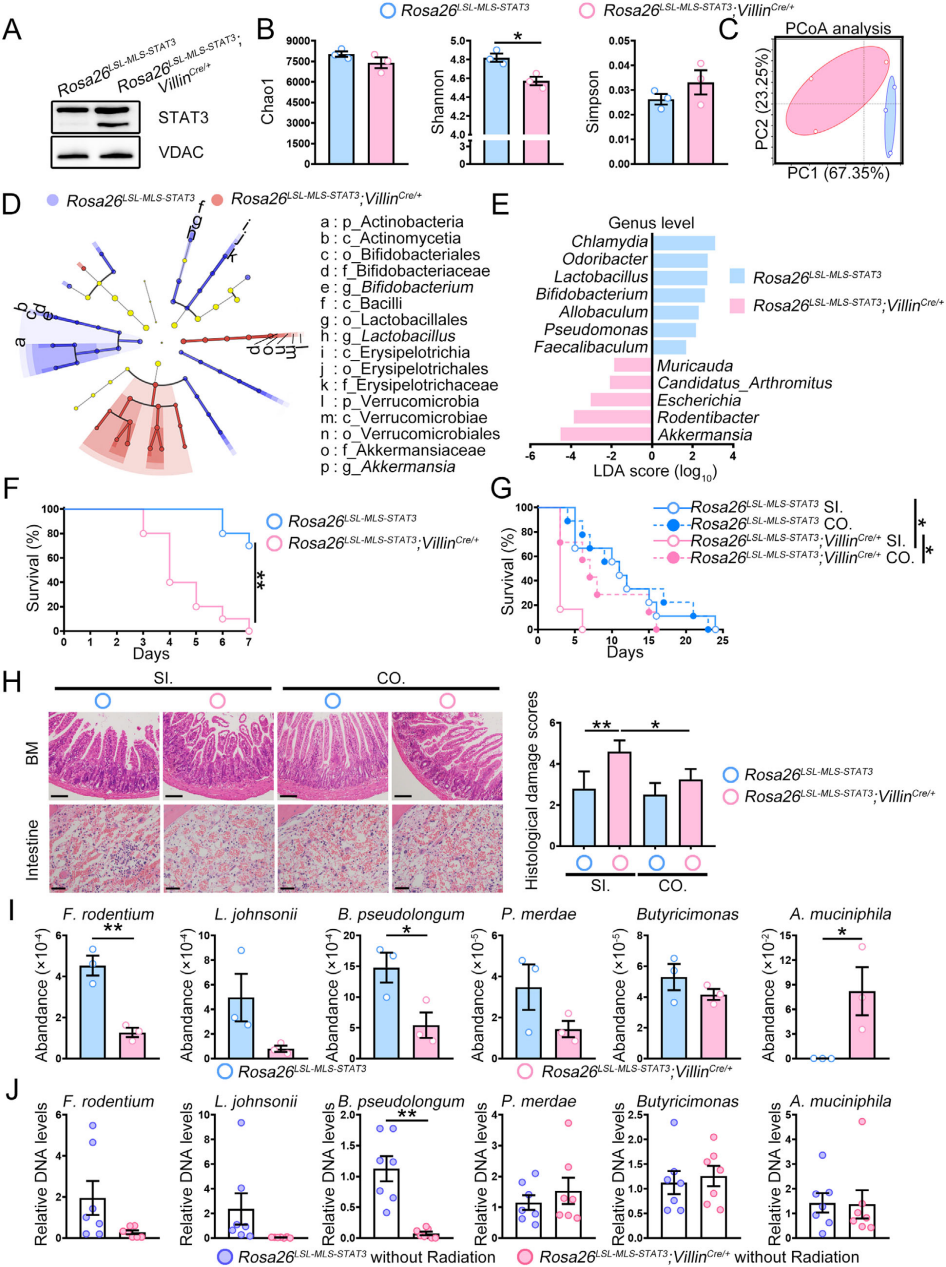
*F. rodentium* abundance was reduced. A) Expression of the indicated proteins in the mitochondrial fraction of epithelial cells was analyzed. Voltage‐dependent anion channel (VDAC) was shown as a loading control. The data represented one of two independent experiments. B) Mice were treated with 7.5 Gy. The metagenomics sequencing was performed 3 days post‐irradiation. A plot of the Shannon–Wiener diversity index, Simpson index, and Chao 1 index was shown. C) Principal coordinate analysis (PCoA) score plot was performed. D) A cladogram representation of taxa enriched in *Rosa26^LSL‐MLS‐mSTAT3^* mice microbiota (blue) and taxa enriched in *Rosa26^LSL‐MLS‐mSTAT3^*;*Villin^Cre/+^* mice microbiota (red). E) Linear discriminant analysis (LDA) scores were analyzed at the genus level. B–E) The figures shown represent a single experiment. *n* = 3 mice/group. F) Kaplan‐Meier survival curves for *Rosa26^LSL‐MLS‐mSTAT3^* mice (*n* = 10) and *Rosa26^LSL‐MLS‐mSTAT3^*;*Villin^Cre/+^* mice (*n* = 10) treated with 7.5 Gy. The figures shown represent a single experiment. G) *Rosa26^LSL‐MLS‐mSTAT3^* mice (alone: *n* = 9; co‐housed: *n* = 9) and *Rosa26^LSL‐MLS‐mSTAT3^*;*Villin^Cre/+^* mice (alone: *n* = 6; co‐housed: *n* = 7) bred alone or co‐housed were treated with 7.5 Gy. Kaplan‐Meier survival curves were analyzed. The figures shown represent a single experiment. H) HE staining of the intestine (scale bar = 100 µm) and BM (scale bar = 50 µm), and damage score of the intestine from *Rosa26^LSL‐MLS‐mSTAT3^* mice (alone: *n* = 5; co‐housed: *n* = 4) and *Rosa26^LSL‐MLS‐mSTAT3^*;*Villin^Cre/+^* mice (alone: *n* = 5; co‐housed: *n* = 4) were analyzed. The data is representative of one of two independent experiments. I) The relative abundance of gut microbiota from *Rosa26^LSL‐MLS‐mSTAT3^* mice and *Rosa26^LSL‐MLS‐mSTAT3^*;*Villin^Cre/+^* mice 3 days post‐irradiation. The figures shown represent a single experiment. *n* = 3 mice per group. J) The relative abundances of indicated bacteria species before irradiation were assessed by real‐time PCR (*n* = 7 per group). The data shown is representative of the combination of two independent experiments. ^*^ *p *< 0.05, ^**^ *p *< 0.01.

To verify the roles of mitochondrial STAT3 in the villi and crypt epithelia influencing gut microbiota and to delineate microbiota‐specific effects from direct mitochondrial STAT3‐mediated impact on HSC, we generated intestine‐specific mitochondrial STAT3 knock‐in mice (*Rosa26^mSTAT3/mSTAT3^;Villin^Cre/+^*) (**Figure** **1**A). Unirradiated *Rosa26^mSTAT3/mSTAT3^;Villin^Cre/+^* mice showed distinct α‐diversity and β‐diversity, along with lesser relative abundances of the probiotic than the unirradiated *Rosa26^mSTAT3/mSTAT3^* mice did (Figure S3A–E, Supporting Information). Post‐IR analysis revealed significant microbiota restructuring in *Rosa26^mSTAT3/mSTAT3^;Villin^Cre/+^* mice, with decreased *Bifidobacterium*, *Lactobacillus*, and *Faecalibaculum* but increased *Akkermansia* abundance (Figure 1B–E). Irradiated *Rosa26^mSTAT3/mSTAT3^;Villin^Cre/+^* mice showed decreased survival under non‐sterile conditions (Figure 1F). Cohousing normalizes microbial communities between genotypes.^[^ ^20^ ^]^ *Rosa26^mSTAT3/mSTAT3^;Villin^Cre/+^* mice cohoused with *Rosa26^mSTAT3/mSTAT3^* mice showed extended survival (Figure 1G), attenuated crypt‐villus damage, and improved BM cellularity (Figure 1H), indicating that STAT3 localization in the mitochondria of villi and crypt epithelia affects the gut microbiome. Thus, the findings indicate that the mitochondrial STAT3 knock‐in mouse model could serve as a tool for selecting potential probiotics essential for IR protection.

To screen for the potential key species, we performed species‐level metagenomics sequencing analysis. *Rosa26^mSTAT3/mSTAT3^;Villin^Cre/+^* mice showed enrichment of *Akkermansia muciniphila* (*A. muciniphila*) but decreased relative abundances of *Faecalibaculum rodentium* (*F. rodentium*), *Lactobacillus johnsonii* (*L. johnsonii*), *Bifidobacterium pseudolongum* (*B. pseudolongum*), *Parabacteroides merdae* (*P. merdae*), and *Butyricimon*as (Figure 1I). The relative abundances of *F. rodentium*, *L. johnsonii*, and *B. pseudolongum* were rescued when *Rosa26^mSTAT3/mSTAT3^;Villin^Cre/+^* mice were cohoused with *Rosa26^mSTAT3/mSTAT3^* mice (Figure S3F, Supporting Information). The relative abundances of *F. rodentium*, *L. johnsonii*, and *B. pseudolongum* decreased in *Rosa26^mSTAT3/mSTAT3^;Villin^Cre/+^* mice that had not been irradiated (Figure 1J). Conversely, *Butyricimonas*, *P. merdae*, and *A. muciniphila* levels were barely affected in unirradiated *Rosa26^mSTAT3/mSTAT3^;Villin^Cre/+^* mice (Figure 1J), indicating that *F. rodentium*, *L. johnsonii*, and/or *B. pseudolongum* may be important in alleviating IR‐induced damage.

### 2.2 F. *rodentium* Mitigated IR‐Induced Injuries in Mice

**Figure 2:**
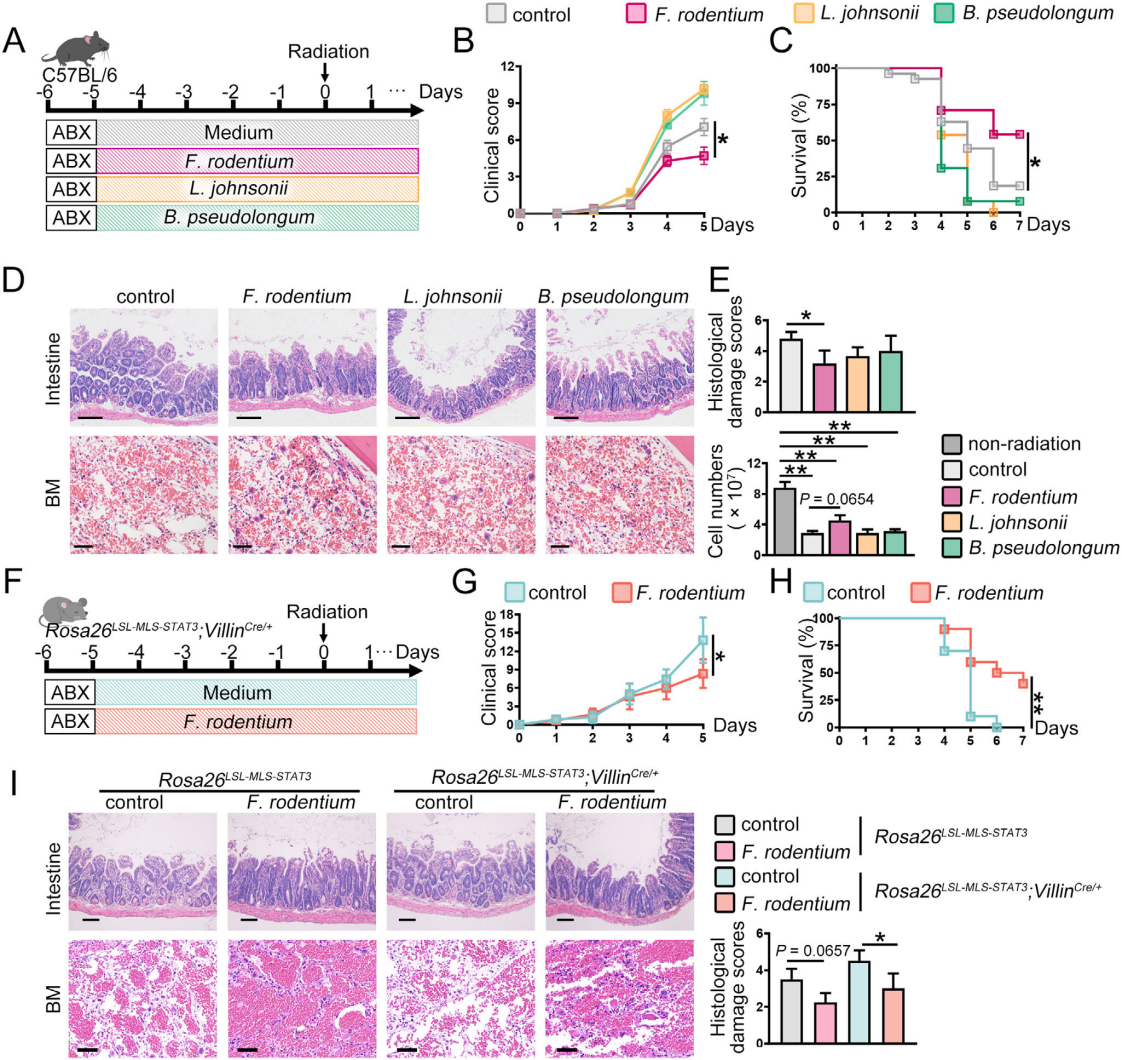
*F. rodentium* alleviates IR‐induced injury in mice. A) WT mice were treated with 7.5 Gy irradiation during the administration of the indicated bacterial strains. B) The clinical score of irradiated mice (Ctrl, *n* = 27; *F. rodentium*, *n* = 24; *L. johnsonii*, *n* = 13; *B. pseudolongum*, *n* = 13) was monitored. C) Kaplan‐Meier survival curves of the irradiated mice (Ctrl, *n* = 27; *F. rodentium*, *n* = 24; *L. johnsonii*, *n* = 13; *B. pseudolongum*, *n* = 13) were analyzed. B, C) The figures show the combination of twice experiments. D) HE staining intestine (scale bar = 100 µm) and BM (scale bar = 50 µm). The figures shown represent one of two independent experiments. E) Cell numbers of BM from untreated mice (*n* = 3) or 1 day post‐irradiated mice (Ctrl, *n* = 5; *F. rodentium*, *n* = 5; *L. johnsonii*, *n* = 3; *B. pseudolongum*, *n* = 3) were counted, and the damage score of the intestine was analyzed. The figures show the combination of twice experiments. F) Mitochondrial STAT3 knock‐in mice were treated with 7.5 Gy irradiation in the administration of either culture medium or *F. rodentium*. G) The clinical score of irradiated mice (*n* = 10 mice per group) was monitored. H) Kaplan‐Meier survival curves of the irradiated mice (*n* = 10 mice per group) were analyzed. (G‐H) The figures show the combination of twice experiments. I) HE staining of the intestine (scale bar = 100 µm) and BM (scale bar = 50 µm) was performed, and the damage score of the intestine was assessed. The figures shown represent one of two independent experiments (*n* = 4 mice per group). ^*^ *p*<0.05; ^**^ *p*<0.01.

To verify which species were key to alleviating IR‐induced damage, we pretreated C57BL/6 mice with either the selected strain or bacterial growth medium by oral gavage for 5 days, followed by exposure to 7.5 Gy IR (**Figure** **2**A). Under non‐sterile conditions, the mice administered *F. rodentium* showed the greatest improvement in clinical scores and survival rates (Figure 2B,C). The mice inoculated with *L. johnsonii* or *B. pseudolongum* did not show better outcomes than those administered the bacterial growth medium (Figure 2B,C). The crypt villi damaged by IR recovered in the presence of *F. rodentium* (Figure 2D,E). Compared to mice administered bacterial growth medium, those that had been administered *F. rodentium* showed attenuation of the IR‐induced decrease in BM cellularity (Figure 2D). Additionally, the nucleated cell count in the BM, which was reduced by IR treatment, was partially rescued in mice treated with *F. rodentium* (Figure 2E). In contrast, *L. johnsonii* and *B. pseudolongum* barely increased the nucleated cell counts (Figure 2E). *F. rodentium* treatment reduced the clinical score and prolonged the survival rate of IR‐treated mitochondrial STAT3 knock‐in mice, along with restoring crypt‐villus damage and improving BM cellularity (Figure 2F–I). These findings showed that *F. rodentium* is critical for mitigating IR‐induced damage.

### 2.3 The *F. rodentium* Metabolite Butyrate was Important for Alleviating IR‐Induced Damage

**Figure 3:**
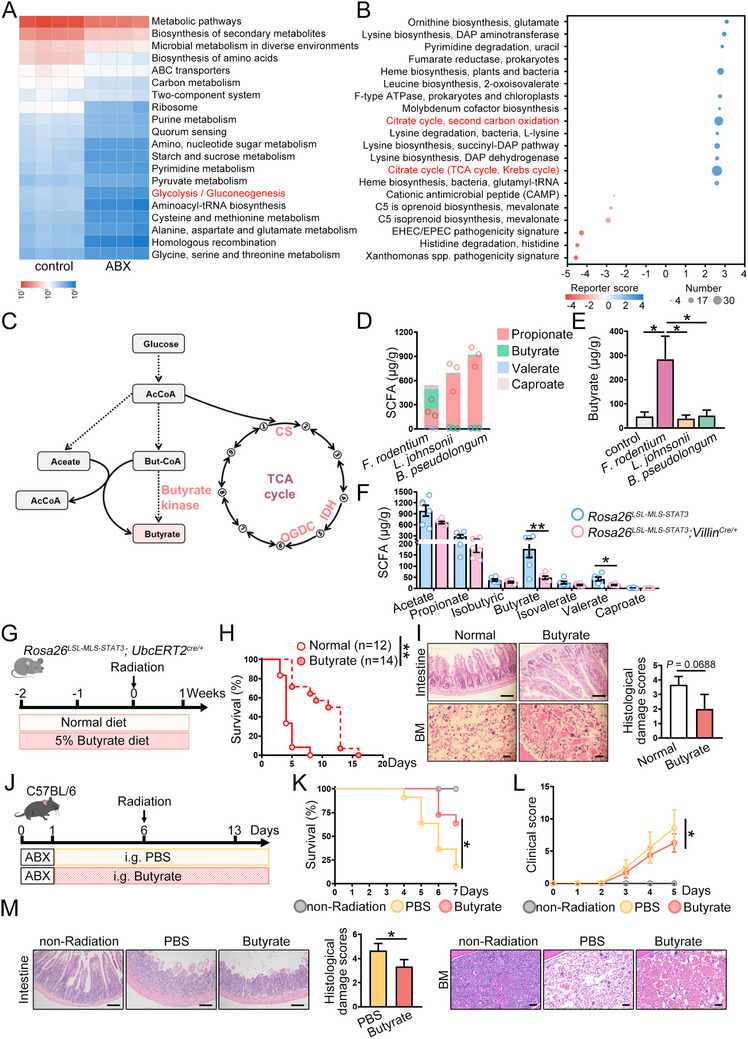
*F. rodentium*‐derived butyrate alleviates IR‐induced injury. A) Microbial function prediction was performed using PICRUSt2 based on the KEGG database. B) KEGG module enrichment analysis of microbial function. **C)** The process of butyrate production. D) The concentrations of SCFAs in bacterial culture medium were examined (*n* = 3 samples per group). E) The concentration of butyrate was examined in irradiated mice treated with the indicated bacterial strains (*n* = 3 mice per group). F) The concentrations of indicated SCFAs were examined in irradiated mice (*n* = 6 mice per group). A–F) The figures shown represent a single experiment. G) The *Rosa26^LSL‐MLS‐mSTAT3^*;*Ubc^ERT2Cre/+^* mice were treated with either normal chow or food containing 5% butyrate. H) Kaplan‐Meier survival curves for irradiated *Rosa26^LSL‐MLS‐mSTAT3^*;*Ubc^ERT2Cre/+^* mice fed with **a** normal diet (*n* = 12 mice) or a sodium butyrate diet (*n* = 14 mice). The figures show the combination of twice experiments. I) HE staining for intestine (scale bar = 100 µm) and BM (scale bar = 50 µm) from irradiated *Rosa26^LSL‐MLS‐mSTAT3^*;*Ubc^ERT2Cre/+^* mice fed with **a** normal diet or sodium butyrate diet (*n* = 3 mice per group). The damage score of the intestine was analyzed. The figures show one of the independent experiments. J) WT mice were treated with PBS or butyrate, followed by irradiation. K) Kaplan‐Meier survival curves for irradiated WT mice treated with PBS or butyrate (*n* = 11 mice per group). L) The clinical score of irradiated mice was monitored (*n* = 11 mice per group). M) HE staining for intestine (scale bar = 100 µm) and BM (scale bar = 50 µm) from irradiated mice treated with PBS or butyrate (*n* = 3 mice per group), and the damage score of the intestine was assessed. K–M) The figures shown represent a single experiment. ^*^ *p*<0.05; ^**^ *p*<0.01.

Metabolic prediction revealed that the metabolic changes were associated with the TCA cycle and glycolysis, along with increased abundance of enzymes important for the TCA cycle (**Figure** **3A,B**; Figure S4A, Supporting Information). Butyrate, a short‐chain fatty acid (SCFA), is produced from carbohydrates via glycolysis (Figure 3C) and presents a major metabolic product of *F. rodentium* (Figure 3D). Butyrate levels increased in mice treated with *F. rodentium* after IR, similar to the findings for the *F. rodentium* culture medium (Figure 3E). The butyrate concentration was lower in *Rosa26^mSTAT3/mSTAT3^;Villin^Cre/+^* mice, in parallel with decreased butyrate kinase abundance (Figure 3F; Figure S4B, Supporting Information). The butyrate kinase abundance positively correlated with *F. rodentium* abundance in *Rosa26^mSTAT3/mSTAT3^* mice and *Rosa26^mSTAT3/mSTAT3^;Villin^Cre/+^* mice (Figure S4C, Supporting Information). Additionally, the abundance of butyrate‐producing bacteria increased in wild‐type mice fed with *F. rodentium* (Figure S4D, Supporting Information), showing that *F. rodentium* could directly or indirectly induce butyrate production and exert radioprotective effects.

Butyrate can control gut homeostasis ^[^ ^22^ ^]^ and regulate the HSC regenerative response.^[^ ^9^ ^]^ To verify whether butyrate is required for *F. rodentium*‐mediated radioprotection, we fed mitochondrial STAT3 knock‐in mice normal chow or food containing 5% butyrate 2 weeks before irradiation and another 2 weeks after irradiation. The irradiated mitochondrial STAT3 mice showed significantly longer lifespan when they were administered butyrate than when they were administered normal chow (Figure 3G,H). The decrease in crypt‐villus units and BM aplasia observed in irradiated mitochondrial STAT3 knock‐in mice was blunted when these mice were fed a butyrate diet (Figure 3I). Butyrate administration also significantly reduced clinical severity scores and prolonged survival in IR‐treated wild‐type mice under non‐sterile conditions; furthermore, it attenuated crypt‐villus architectural damage and improved BM cellularity (Figure 3J–M). These findings showed that *F. rodentium* both directly and indirectly induced butyrate production and alleviated IR‐induced injuries.

### 2.4 F. *rodentium*/Butyrate Promoted Intestinal Integrity and Hematopoietic Recovery

**Figure 4:**
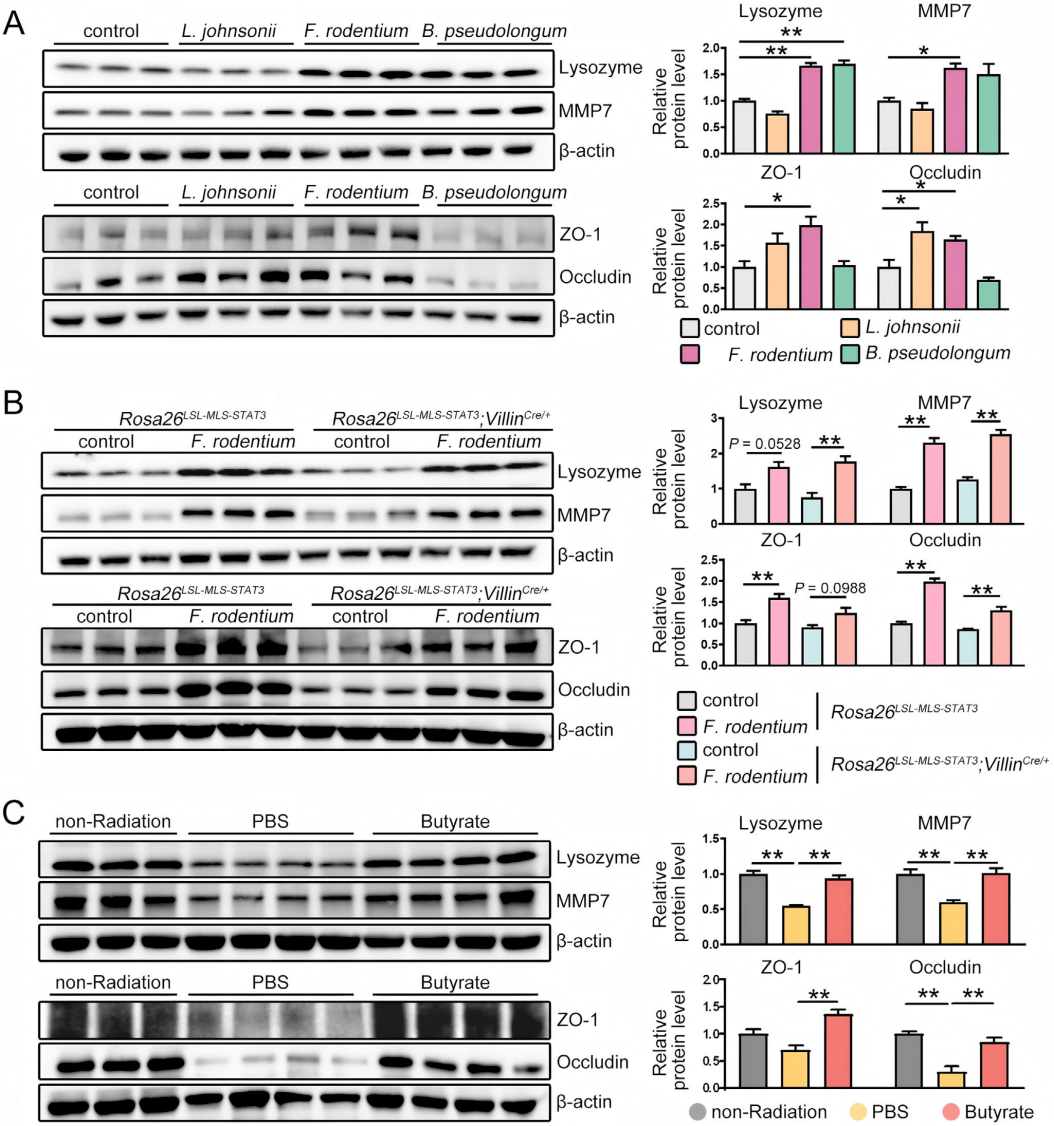
*F. rodentium* and butyrate promote intestinal mucosal repair. A–C) The indicated protein levels were examined by Western blotting. β‐actin was shown as a loading control. The indicated protein levels were quantified by ImageJ software. The data shown are representative of one of two independent experiments. ^*^ *p*<0.05; ^**^ *p*<0.01.

Butyrate modulates intestinal mucosal barrier integrity.^[^ ^23^ ^]^ Consistent with this previous finding, we noted that the levels of MMP7, which is responsible for antimicrobial peptide maturation^[^ ^24^ ^]^ and lysozyme activity, and the tight junction proteins zonula occludens‐1 (ZO‐1) and occludin increased when mice were fed *F. rodentium* or butyrate (**Figure** **4**A–C). This finding indicated that *F. rodentium*/butyrate administration rescued the IR‐induced damage to gut barrier integrity and defensive ability.

**Figure 5:**
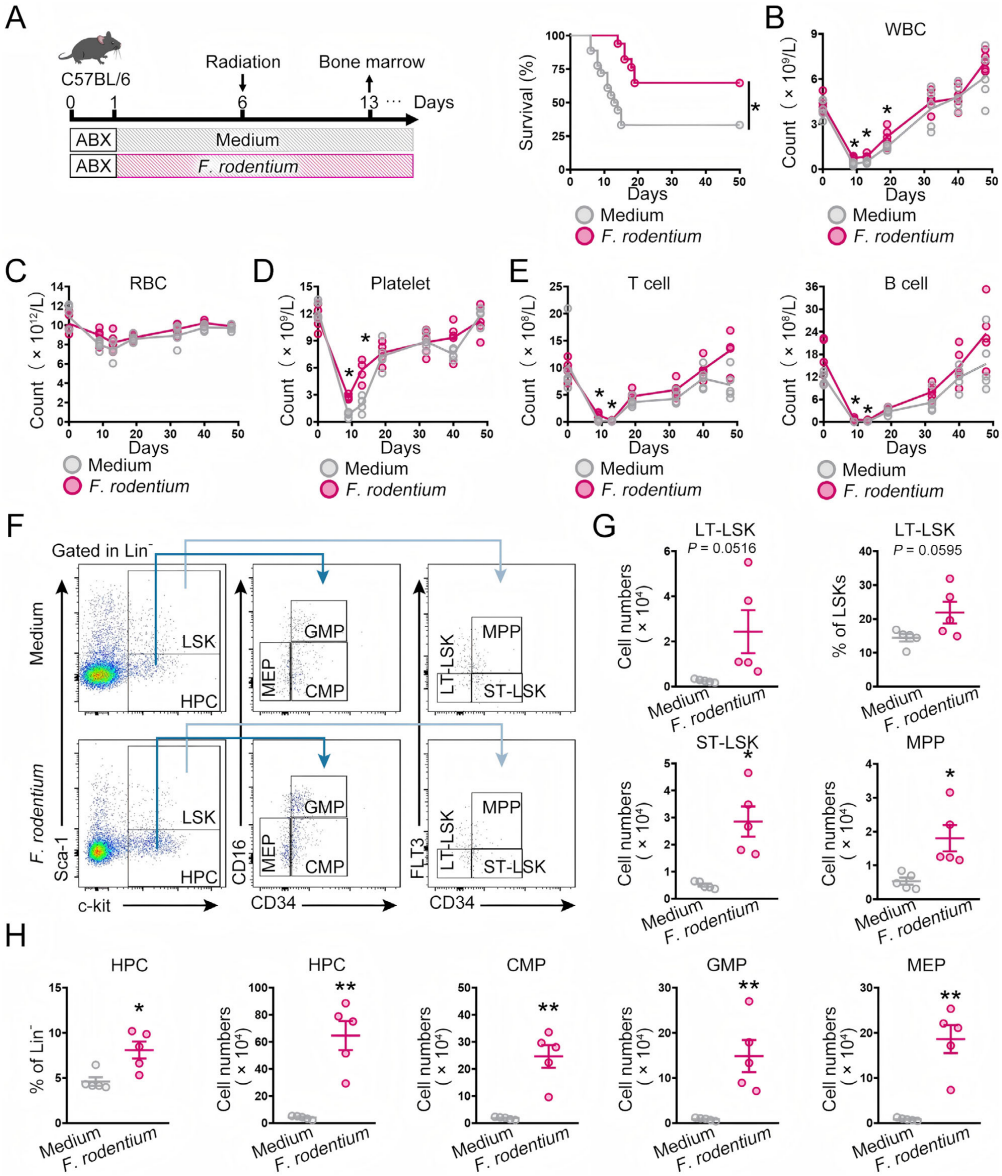
*F. rodentium* promotes hematopoietic recovery. A) WT mice were treated with 5 Gy irradiation in the administration of either culture medium (*n* = 18) or *F. rodentium* (*n* = 17). The survival was monitored. B–E) The counts of WBC, RBC, platelets, T cells, and B cells in the peripheral blood of mice were monitored at the indicated time points (days 0 (pre‐irradiation), 9‐, 13‐, 19‐, 32‐, 40‐, and 48‐days post‐irradiation) before and after 5 Gy total body irradiation. The data shown are representative of one of two independent experiments. F–H) The irradiated mice were treated with culture medium or *F. rodentium*. 13 days after treatment, the indicated subpopulations in BM were analyzed by FACS (*n* = 5 mice per group). Data were visualized in flow cytometry plots (F) and quantitatively analyzed (G, H). The figures shown represent a single experiment. HPC, hemapoietic progenitor cells; LSK, Lin^−^Sca1^+^c‐Kit^+^ cells; LT‐LSK, Long‐term LSK; ST‐LSK, short‐term LSK; GMP, granulocyte‐macrophage progenitors; CMP, common myeloid progenitors; MEP, megakaryocyte‐erythrocyte progenitors; MPP, multipotent progenitors. ^*^ *p*<0.05, ^**^ *p*<0.01.

To assess whether *F. rodentium*/butyrate could promote hematopoietic regeneration, we treated wild‐type mice with a sublethal dose of total body irradiation.^[^ ^25^ ^]^ We found that there were similar drops in cellularity across the bacterial growth medium and *F. rodentium* groups for the first 10 days. However, consistent with the survival data, whole blood count analysis revealed that *F. rodentium* administration accelerated cellularity recovery to a significantly greater extent than the bacterial growth medium alone (**Figure** **5**A–E). On Day 13, *F. rodentium*‐treated mice exhibited an increase in the counts of long‐term (LT) and short‐term (ST) Lin^−^Sca1^+^c‐Kit^+^ (LSK) cells, which are essential for sustained hematopoiesis, and their downstream progenitors, including multipotent progenitors (MPPs), common myeloid progenitors (CMPs), megakaryocyte‐erythrocyte progenitors (MEPs), and granulocyte‐macrophage progenitors (GMPs) (Figure 5F–H). On Day 60, the counts of LT‐LSK cells and their downstream progenitors were higher in mice treated with *F. rodentium* than in those treated with the bacterial growth medium alone (Figure S5A–D, Supporting Information). We also performed competitive BM transplantation and found that BM cells harvested from *Rosa26^mSTAT3/mSTAT3^;Villin^Cre/+^* mice exhibited lesser proliferation than those harvested from *Rosa26^mSTAT3/mSTAT3^* mice in irradiated CD45.1 mice (Figure S5E,F, Supporting Information). Additionally, we found that the colony number and mRNA levels of *Forkhead box O3a* (*FOXO3a*), which regulates HSC self‐renewal ^[^ ^26^ ^],^ in sorted Lin^−^Sca1^+^c‐Kit^+^CD48^−^CD150^+^ cells from *F. rodentium‐*fed mice had increased (Figure S5G,H, Supporting Information). The IR‐induced mRNA expression of the proapoptotic gene *BCL‐2‐associated X protein* (*BAX*), a downstream target of p53, was reversed in the presence of *F. rodentium* (Figure S5G, Supporting Information).

**Figure 6:**
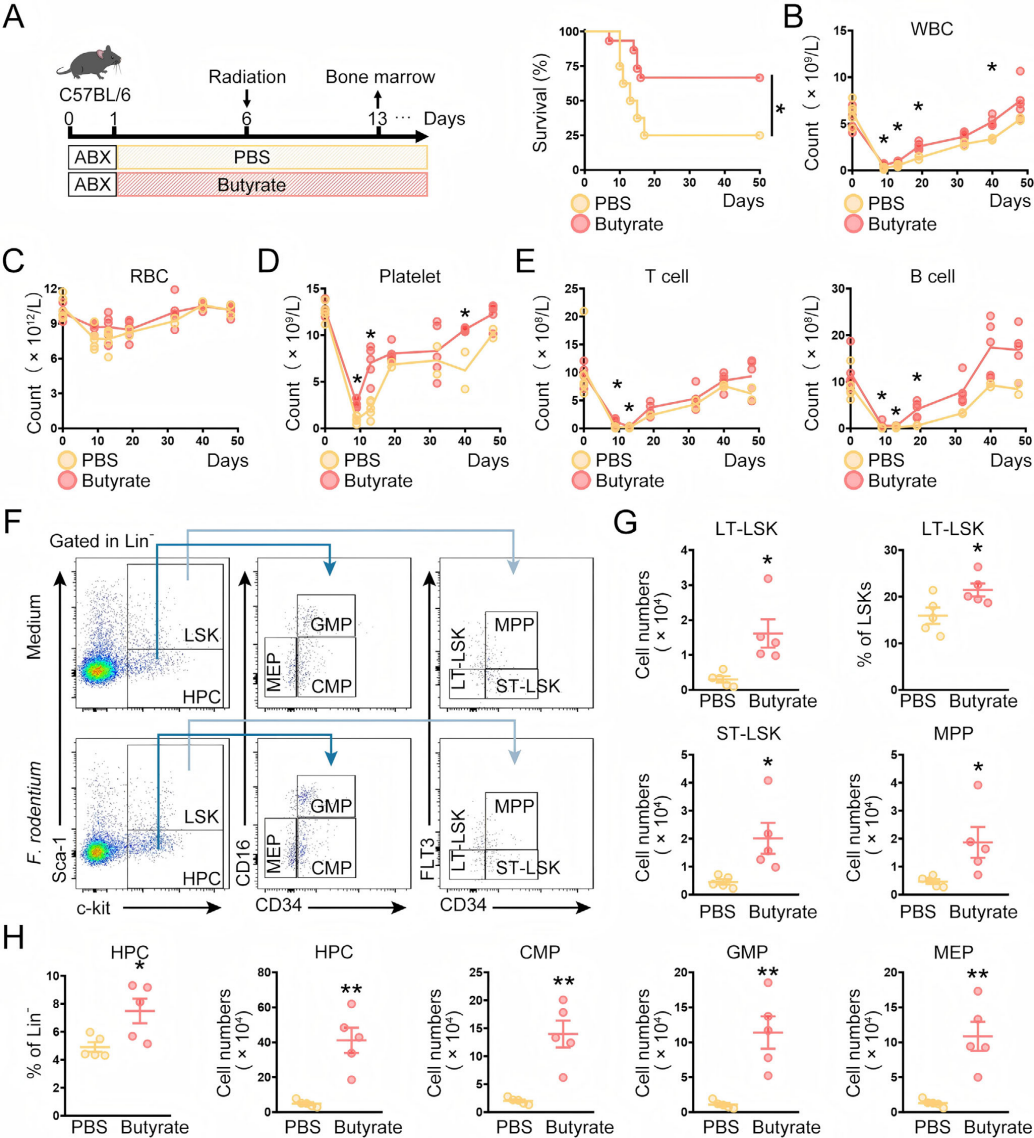
Butyrate accelerates hematopoietic recovery. A) 5 Gy irradiated mice were treated with either PBS (*n* = 8) or butyrate (*n* = 14), and the survival was monitored. B–E) The counts of WBC, RBC, platelets, T cells, and B cells in the peripheral blood of mice were monitored at the indicated time points (days 0 (pre‐irradiation), 9, 13, 19, 32, 40, and 48 days post‐irradiation) before and after 5 Gy total body irradiation. F–H) 13 days post‐irradiation, the indicated subpopulations in the BM were analyzed by FACS (*n* = 5 mice per group). Data were visualized in flow cytometry plots (F) and quantitatively analyzed G, H). The figures shown represent a single experiment. HPC, hemapoietic progenitor cells; LSK, Lin^−^Sca1^+^c‐Kit^+^ cells; LT‐LSK, Long‐term LSK; ST‐LSK, short‐term LSK; GMP, granulocyte‐macrophage progenitors; CMP, common myeloid progenitors; MEP, megakaryocyte‐erythrocyte progenitors; MPP, multipotent progenitors. ^*^ *p*<0.05, ^**^ *p*<0.01.

Similar to the findings for *F. rodentium* administration, butyrate triggered hematopoietic regeneration in vivo (**Figure** **6**A–H), along with an increase in colony numbers and the relative mRNA levels of *FOXO3a* and *Runt‐related transcription factor 1* (*RUNX1*) (Figure S5I,J, Supporting Information). These findings indicated that *F. rodentium* promoted hematopoietic regeneration via its metabolite butyrate.

### 2.5 Inhibition of ERK/PKM2‐Mediated p53 Activity was Indispensable for *F. rodentium*/Butyrate–Induced Decrease in c‐Kit+ Cell Apoptosis

**Figure 7:**
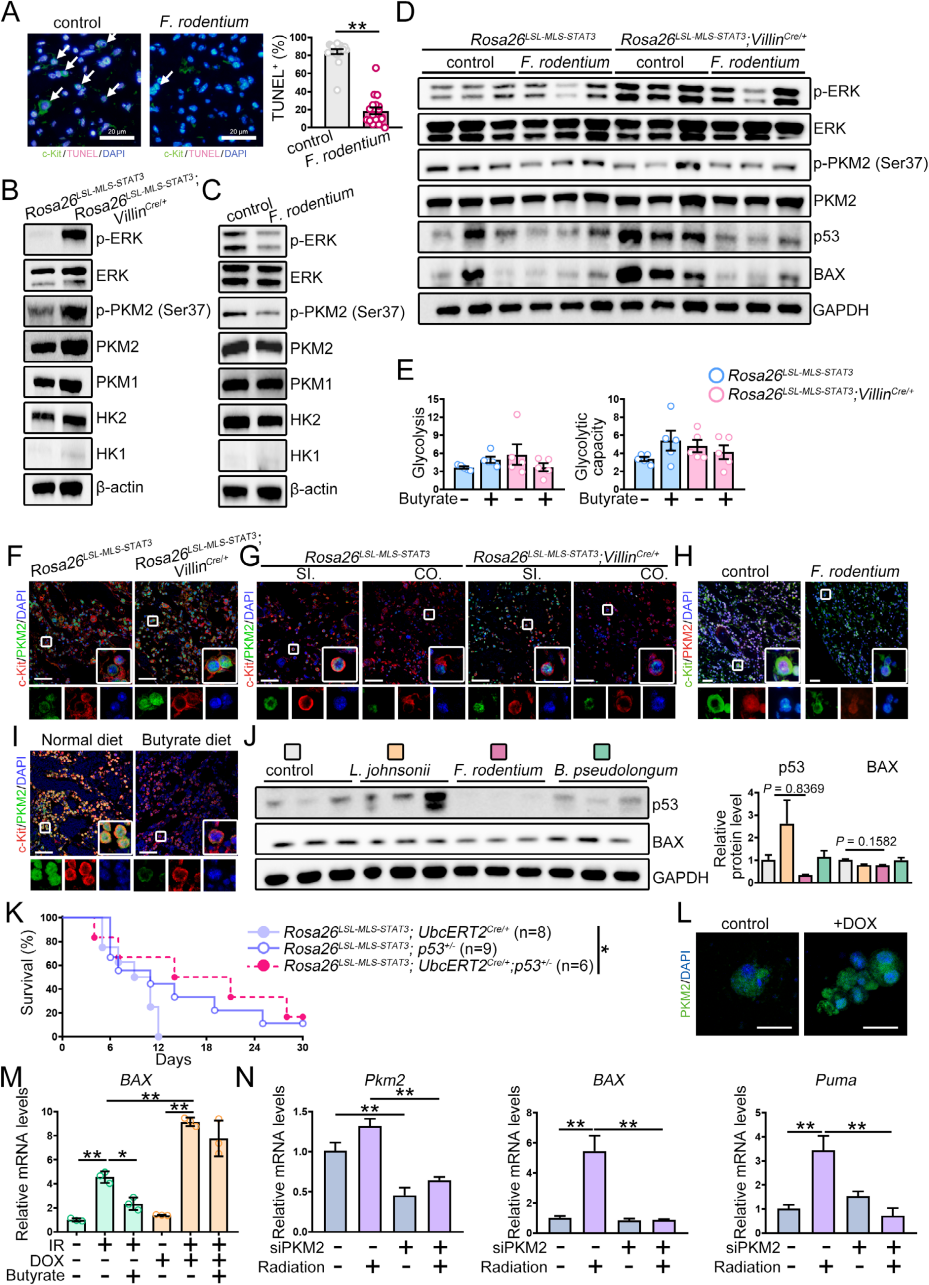
Butyrate attenuated IR‐induced apoptosis of c‐Kit^+^ cells by inhibiting the ERK/PKM2/p53 pathway. A) The numbers of apoptotic c‐Kit^+^ cells isolated from either control or *F. rodentium*‐treated irradiated mice were examined by TUNEL staining. B–D) The indicated protein levels in c‐Kit^+^ cells were examined by Western blotting analysis. The indicated protein levels were quantified by ImageJ software. The data shown are representative of one of two independent experiments. E) The glycolysis and glycolytic capacity of irradiated c‐Kit^+^ cells cultured with or without butyrate were investigated. The figures shown represent a single experiment. F–I) Multiplex immunofluorescence was performed to examine nuclear PKM2 in c‐Kit^+^ cells of irradiated mice treated with the indicated approach (scale bar = 20 µm). Images were obtained under a Leica STELLARIS 5 confocal microscope. The data shown are representative of one of two independent experiments. J) The indicated protein levels in c‐Kit^+^ cells were examined by Western blotting analysis. The indicated protein levels were quantified by ImageJ software. The data shown are representative of one of two independent experiments. K) The survival of irradiated *Rosa26^LSL‐MLS‐Stat3^;p53^±^* (*n* = 8)_,_ irradiated *Rosa26^LSL‐MLS‐mSTAT3^*;*Ubc^ERT2Cre/+^* (*n* = 5) and irradiated *Rosa26^LSL‐MLS‐Stat3^; Ubc^ERT2Cre^;p53^+/−^* mice (*n* = 6) were analyzed by Kaplan‐Meier analysis. The figures show the combination of twice experiments. L) Fetal liver cells from WT mice were infected with nuclear PKM2 lentivirus and treated with or without DOX. 7 days later, immunofluorescence was performed to examine nuclear PKM2 expression (scale bar = 20 µm). Images were obtained under a Leica STELLARIS 5 confocal microscope. The figures shown represent a single experiment. M) The relative mRNA levels of *BAX* in nuclear PKM2 lentivirus‐infected fetal liver cells cultured with the indicated treatment were examined. The data shown are representative of one of two independent experiments. N) Real‐time RT‐PCR was used to examine the mRNA levels of *Pkm2*, *BAX*, and *Puma* in c‐Kit^+^ cells treated with negative control siRNA or siPKM2 in the presence or absence of 5 Gy irradiation. The data shown are representative of one of two independent experiments. ^*^ *p*<0.05, ^**^ *p*<0.01.

Given that BM hematopoietic cells are progenitors of diverse blood lineages and are sensitive to IR‐induced apoptosis, we examined the effects and underlying mechanisms by which *F. rodentium*/butyrate attenuated IR‐triggered apoptotic signaling in c‐Kit^+^ hematopoietic progenitors. *F. rodentium* treatment was found to lead to a decrease in apoptotic c‐Kit^+^ cells (**Figure** **7**A).

IR activates ERK, which promotes compensatory glycolysis, thereby ensuring energy provision after irradiation.^[^ ^27^ ^]^ Butyrate suppresses ERK activation.^[^ ^28^ ^]^ Therefore, we examined ERK activation and the enzymes involved in glycolysis and found that the p‐ERK level increased in c‐Kit^+^ cells sorted from IR‐treated conditional mitochondrial STAT3 mice, whereas the p‐ERK level was decreased by *F. rodentium* (Figure 7B–D; Figure S6A, Supporting Information). The Seahorse assay findings showed that glycolysis and glycolytic capacity slightly increased in sorted c‐Kit^+^ cells, which were barely affected by butyrate (Figure 7E).

ERK binds to and triggers PKM2 phosphorylation, leading to PKM2 nuclear localization.^[^ ^29^ ^]^ p‐PKM2 levels were elevated in mitochondrial STAT3 knock‐in mice lacking *F. rodentium* and butyrate; this elevation was reversed in the presence of *F. rodentium* (Figure 7B–D; Figure S6B, Supporting Information). The protein level of PKM2, but not those of HK1/2 or PKM1, was slightly increased in mitochondrial STAT3 knock‐in mice, while it slightly decreased after *F. rodentium* treatment (Figure 7B,C; Figure S6C, Supporting Information). Upon IR treatment, the mRNA levels of the genes involved in glycolysis were almost identical in the c‐kit^+^ cells from *Rosa26^mSTAT3/mSTAT3^;Villin^Cre/+^* mice to those from *Rosa26^mSTAT3/mSTAT3^* mice (Figure S6D, Supporting Information). PKM2 levels were higher in the nuclei of c‐Kit^+^ cells from IR‐treated *Rosa26^mSTAT3/mSTAT3^;Villin^Cre/+^* mice than in those from IR‐treated *Rosa26^mSTAT3/mSTAT3^* mice (Figure 7F). Nuclear PKM2‐positive c‐Kit^+^ cells decreased when conditional mitochondrial STAT3 knock‐in mice were cohoused with *Rosa26^mSTAT3/mSTAT3^* mice (Figure 7G). Additionally, both *F. rodentium* and butyrate reduced nuclear PKM2 expression in c‐Kit^+^ cells from IR‐treated wild‐type mice (Figure 7H,I).

PKM2 translocates to the nucleus to determine cell fate by binding to numerous transcription factors such as p53.^[^ ^30^ ^]^ To further explore the role of nuclear PKM2 in c‐Kit^+^ cells, we examined apoptotic proteins and found that the p53 and BAX protein levels decreased in c‐Kit^+^ cells from mitochondrial STAT3 knock‐in mice administered *F. rodentium* (Figure 7D; Figure S6E, Supporting Information). Similarly, the p53 and BAX protein levels were reduced in c‐Kit^+^ cells isolated from *F. rodentium*‐fed wild‐type mice (Figure 7J). Moreover, deletion of one allele of the *P53* gene significantly extended the lifespan of irradiated mice (Figure 7K).

We ectopically expressed nuclear PKM2 and found that it potentiated IR‐induced *BAX* expression, which was not rescued by butyrate (Figure 7L,M). PKM2 knockdown decreased the IR‐induced *BAX* and *Puma* mRNA expression (Figure 7N). These findings indicated that, upon IR treatment, ERK‐induced PKM2 nuclear localization potentiated p53 transcriptional activity, leading to c‐Kit^+^ progenitor apoptosis, which could be attenuated by *F. rodentium* via its metabolite butyrate.

### 2.6 *Holdemania biformis* Abundance Decreased in Individuals with Leukemia

**Figure 8:**
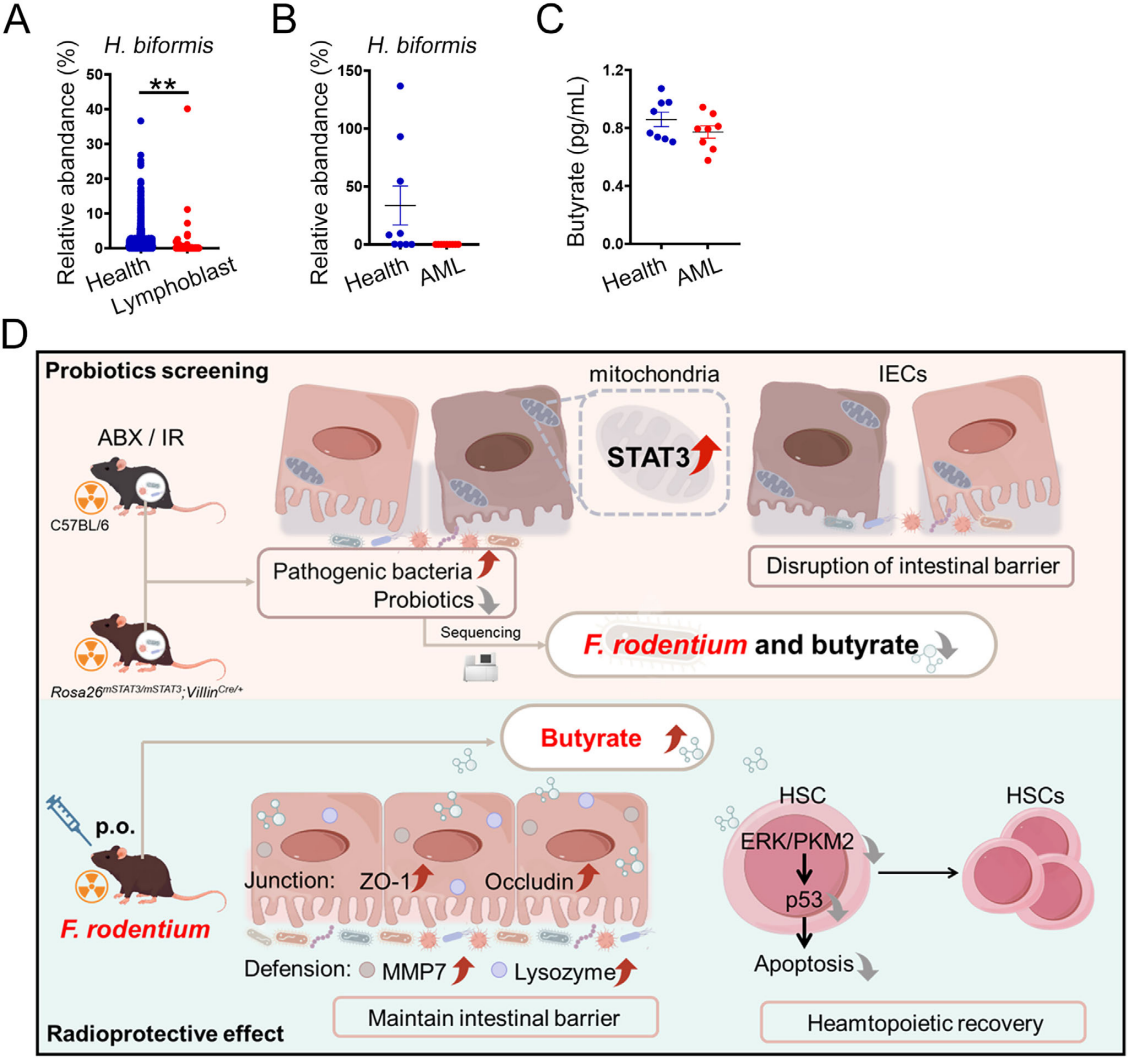
*H. biformis* had potential value in hematology. A) The relative abundance of *H. biformis* was assessed in healthy volunteers (*n* = 4901) and patients who suffered from precursor cell lymphoblastic leukemia‐lymphoma (*n* = 251) by utilizing the GMrepo database (https://gmrepo.humangut.info). ^**^ *p*<0.01. B) The relative abundance of *H. biformis* was assessed in healthy volunteers and AML patients (*n* = 9 per group). C) The concentrations of butyrate were assessed by LC‐MS (*n* = 8 per group). D) The model showed the probiotics screening and underlying mechanisms of *F. rodentiumn* in alleviating IR‐induced tissue injury. Stress‐induced STAT3 mitochondria localization decreases *F. rodentium* abundance and its metabolite butyrate concentration; Supplementation of *F. rodentium* or butyrate increases the protein levels of tight junction protein and defensive factors, blocks ERK/PKM2/p53 signaling‐mediated apoptosis of hematopoietic progenitors, reinforcing intestinal integrity and promoting hematopoietic recovery, which ultimately prolongs mice survival.

Gut microbiota diversity and butyrate concentrations decrease in patients with graft‐vs‐host disease (GVHD).^[^ ^31^ ^]^ *Holdemanella biformis*, the human homologue of *F. rodentium*, belongs to the genus *Holdemania*. Its abundance was reduced in patients with precursor cell lymphoblastic leukemia–lymphoma or acute myeloid leukemia (AML; **Figure** **8**A,B). Moreover, the butyrate concentration tended to decrease in the patients with AML (Figure 8C), highlighting the potential importance of probiotics, such as *Holdemanella biformis*, and gut microbiota metabolites, including butyrate, in HSC maintenance and leukemogenesis.

Taken together, our findings showed that, under stress conditions, mitochondrial STAT3 decreased the abundance of *F. rodentium* and its metabolite butyrate. *F. rodentium* treatment increased butyrate levels, restored gut barrier integrity by upregulating the levels of tight junction proteins (ZO‐1 and occludin) and defense factors (e.g., MMP7 and lysozyme), enhanced hematopoietic recovery, and suppressed nuclear PKM2/p53 signaling–mediated apoptosis of c‐Kit^+^ cells, and thus mitigated IR‐induced tissue damage and prolonged mouse survival (Figure 8D). Collectively, these findings establish *F. rodentium*/butyrate as a promising microbiota‐directed therapeutic strategy for radiation injury.

## 3 Discussion

To establish a murine model enabling screening of the key probiotic strain(s) critical for radioprotection, we performed mitochondrial proteomic profiling in IECs and observed STAT3 accumulation within intestinal mitochondria. We identified the probiotic *F. rodentium* by utilizing mitochondrial STAT3 knock‐in mice. Moreover, we have demonstrated that *F. rodentium* and its metabolite butyrate confer radioprotection by enhancing intestinal barrier integrity and facilitating hematopoietic recovery.

During the first few weeks after allogeneic HSC transplantation, the decrease in intestinal diversity of the gut microbial community could increase the risk of acute GVHD‐related mortality and disease relapse.^[^ ^32^, ^33^ ^]^ Additionally, high intestinal diversity of the gut microbial community is associated with reduced overall mortality in transplant patients.^[^ ^34^ ^]^ Utilizing ampicillin to delete *Lactobacillus* species has been found to worsen GVHD, while replenishing *Lactobacilli* was found to protect mice from GVHD.^[^ ^35^, ^36^ ^]^ Moreover, *Enterococcus* expansion promoted by the common nutrient lactose exacerbates GVHD severity.^[^ ^37^ ^]^ Similarly, in meropenem‐induced expansion of *Bacteroides thetaiotaomicron*, the expression of enzymes critical for mucin glycan degradation was found to be upregulated, increasing the risk of intestinal GVHD.^[^ ^38^ ^]^ A high‐fat diet induces gut microbiome dysbiosis, impairs BM niche function, decreases LT‐LSK cell counts, and causes a shift from lymphoid to myeloid cell differentiation.^[^ ^39^ ^]^ The Lachnospiraceae family strains and nicotinamide riboside can alleviate IR‐induced injury by promoting GI repair and accelerating hematopoietic recovery.^[^ ^10^, ^11^ ^]^ These findings highlight the importance of microbial balance in mediating hematopoiesis and radioprotection. However, identification of specific bacterial species that exert a radioprotective effect from the vast microbial community of the GI tract poses significant challenges.

Mitochondrial alterations in IECs can affect gut microbiota composition. In the current study, we performed mitochondrial proteomic profiling and showed that, under stress conditions, STAT3 was localized in IEC mitochondria (Table S1 and Figure S1, Supporting Information). STAT3 is a transcription factor predominantly activated by tyrosine 705 (Y705) or serine 727 (S727) phosphorylation, with distinct functional roles attributed to p‐STAT3 (Y705) and p‐STAT3 (S727).^[^ ^40^ ^]^ Y705 phosphorylation promotes STAT3 translocation to the nucleus, where it transcriptionally regulates proliferation‐ and survival‐related genes.^[^ ^41^, ^42^, ^43^ ^]^ Nuclear STAT3 may influence gut microbiota by controlling the expression of antimicrobial peptides. IL‐22, produced by T helper 17 (Th17) cells, group 3 innate lymphoid cells (ILC3s), and γδT cells, enhances the expression of antimicrobial peptides by triggering p‐STAT3 (Y705) in IECs. ^[^ ^44^, ^45^ ^]^ In addition, nuclear STAT3 activation affects some bacteria clearance via regulating the expression of autophagy‐related proteins.^[^ ^46^ ^]^ Our group and other research groups have shown that stimulus‐induced phosphorylation at S727 facilitates translocation of STAT3 to the mitochondria, where it modulates mitochondrial functions such as metabolic reprogramming of biosynthetic precursors and cellular activity.^[^ ^21^, ^47^ ^]^ Therefore, we hypothesized that the mitochondrial STAT3 of IECs influences gut microbial ecology. Mitochondrial STAT3 in the villi and crypt epithelium was found to alter the gut microbiome composition, which was correlated with a decrease in mouse survival and crypt‐villus integrity and hematopoietic depletion (Figure 1A–H; Figure S3A–E, Supporting Information).

Mitochondrial STAT3 regulates the production of reactive oxygen species (ROS) ^[^ ^48^ ^],^ which compromise gut barrier integrity and impair hematopoietic progenitor cell function.^[^ ^49^, ^50^, ^51^ ^]^ Consistent with this finding, our results showed that ROS levels increased in IECs isolated from irradiated mitochondrial STAT3 knock‐in mice (Figure S7A, Supporting Information). *F. rodentium* administration suppressed ROS accumulation, ameliorated radiation‐induced tissue damage, and prolonged mouse survival (Figure S7B, Supporting Information; Figure 2). These findings suggested that *F. rodentium* attenuated mitochondrial STAT3–driven ROS production, potentially contributing to its protective effects against radiation‐compromised gut integrity and hematopoietic recovery. However, the precise mechanisms underlying *F. rodentium*‐mediated inhibition of mitochondrial STAT3‐regulated ROS production require further investigation.

SCFAs are metabolized by the gut microbiota during the fermentation of partially digestible and nondigestible polysaccharides in the distal intestine.^[^ ^52^ ^]^ SCFAs, particularly butyrate, constitute some of the key metabolites of *F. rodentium* (Figure 3D–F).^[^ ^53^ ^]^ We found that feeding mice with *F. rodentium* also increased the abundance of butyrate‐producing bacteria such as *E. rectale* (Figure S4D, Supporting Information), suggesting that *F. rodentium* directly and/or indirectly enhances butyrate production. Administering *F. rodentium* or butyrate to mice extended the lifespan of IR‐treated mice (Figures 2 and 3), in contrast to previous research showing that propionate, but not butyrate, ameliorated IR‐induced damage.^[^ ^10^ ^]^ This discrepancy likely reflects the differences between the mouse models and treatment protocols used in the two studies. In Guo et al.’s study, *Lachnospiraceae* was isolated from radiation‐resistant mice; the irradiated mice were then treated with *Lachnospiraceae* via twice‐weekly oral gavage or with 200 mm propionate administered in drinking water to evaluate their radioprotective efficacy.^[^ ^10^ ^]^ In contrast, our study screened *F. rodentium* by utilizing mitochondrial STAT3 transgenic mice, and verified their radioprotection effects by daily oral administration of *F. rodentium* or 20 mg kg^−1^ butyrate to irradiated mice, which could collectively account for the observed discrepancies in radioprotective outcomes.

Butyrate exerts several effects on host metabolism and the immune system.^[^ ^52^ ^]^ It regulates the HSC regenerative response ^[^ ^9^ ^]^ and suppresses the expression and activity of sirtuin1 (SIRT1), a member of the class III histone deacetylase family,^[^ ^54^ ^]^ thereby alleviating IR‐induced GI stem cell death in mice.^[^ ^55^ ^]^ In the current study, we found that *F. rodentium* and butyrate modulate intestinal integrity and promote hematopoietic regeneration (Figures 4, 5, 6; Figure S5A–F, Supporting Information). Furthermore, *F. rodentium* and butyrate reversed the IR‐induced inhibition of c‐Kit^+^ cell proliferation and restored *FOXO3a* and *RUNX1* mRNA levels, which had been suppressed by IR in c‐Kit^+^ cells (Figure S5G–J, Supporting Information). Studies have shown that butyrate can exert protective effects in mice when administered either in a diet containing 5% butyrate^[^ ^56^ ^]^ (corresponding to a daily intake of 1.6–2.3 mmol day^−1^ per mouse, assuming a daily food intake of 2.8–4.0 g^[^ ^57^ ^]^) or orally at doses of 15 or 20 mg kg^−1^ (equivalent to 0.0034 or 0.0045 mmol per 20 g mouse, respectively)^[^ ^58^, ^59^ ^]^ Additionally, previous study and our unpublished data have indicated that butyrate increases the clonal expansion of HSCs in a dose‐dependent manner,^[^ ^60^ ^]^ suggesting that its protective effects may also be dose‐dependent.

We also found that the mechanism underlying this inhibition of IR‐induced apoptosis of c‐Kit^+^ cells by *F. rodentium* and its metabolite butyrate involved abolishing activated ERK–driven PKM2 nuclear localization (Figure 7). Although PKM2 is a key glycolytic enzyme, the glycolysis and the glycolytic capacity showed no significant difference in the presence of butyrate (Figure 7E). PKM2 silencing reduced the IR‐induced increase in proapoptotic gene expression (Figure 7N). These data showed that the gut microbiota‐derived metabolite butyrate promotes hematopoietic recovery via multiple mechanisms, including rescuing apoptosis via inhibition of PKM2 nuclear localization.

PKM2 presents as a monomer, dimer, or tetramer.^[^ ^61^ ^]^ It mainly occurs as a monomer or dimer with low enzyme activity, shuttles to the nucleus, and binds to transcription factors (e.g., p53) to regulate gene expression. The tetrameric form has high glycolytic activity and has been found to suppress p53 transcriptional activity in myocardial cells.^[^ ^62^ ^]^ In our current study, we showed that, in IR‐treated c‐Kit^+^ cells, nuclear PKM2 promoted p53‐induced apoptosis (Figure 7L–N). This different effect of PKM2 on p53 function may be attributable to the use of different tissue types.

p53, a tumor suppressor, serves as a key transcription factor that regulates apoptosis, cell cycle, metabolism, and HSC self‐renewal.^[^ ^63^ ^]^ Under stress conditions, p53 is rapidly activated to execute its biological functions. In the current study, we found that, in the absence of *F. rodentium* and butyrate, the protein levels of p53 and its downstream target BAX significantly increased in c‐Kit^+^ cells isolated from irradiated mice (Figure 7D,J). Butyrate suppressed p53 transcriptional activity by inhibiting nuclear PKM2 accumulation (Figure 7M), suggesting that the decrease in butyrate‐producing bacteria and butyrate levels due to IR compromised gut homeostasis by impairing gut barrier integrity. When butyrate levels were deficient, IR‐activated ERK2 promoted PKM2 nuclear localization, thereby enhancing p53 transcriptional activity in HSCs and ultimately leading to irradiation‐associated lethality (Figure 8D).

This study had several limitations. Although mitochondrial STAT3 induced microbiome dysfunction, we cannot exclude the possibility that endogenous nuclear STAT3 may cause gut microbiota dysbiosis. Furthermore, the gut microbial ecosystem is highly dynamic and complex. There is no clear evidence indicating that *F. rodentium* directly modulates the abundances of butyrate‐producing bacterial strains. Furthermore, the underlying mechanisms by which *F. rodentium* decreased mitochondrial STAT3‐mediated ROS remain to be fully elucidated. Finally, the abundances of *H. biformis*, the human homologue of *F. rodentium*, were reduced in AML patients. Owing to the limited sample size, the patient data should be interpreted with caution. Further researches involving larger cohorts of patients are needed to validate *H. biformis* relevance.

## 4 Conclusion

Our results demonstrated that *F. rodentium* and its metabolite butyrate alleviated IR‐induced tissue damage by improving intestinal barrier integrity and hematopoietic recovery, revealing a potential therapeutic strategy against radiation injury.

## 5 Experimental Section

### Cells

HEK‐293T (293T) cells were purchased from FuHeng (FuHeng Cell Center, Shanghai, China) and cultured in DMEM (BC‐M‐005, Bio‐Channel, Nanjing, China) supplemented with 10% fetal bovine serum (FBS) (#3022A, Umedium, Hefei, China) and 1% penicillin and streptomycin (100 µg mL^−1^; 15140‐122, Gibco, USA).

Hematopoietic progenitor cells enriched from fetal liver cells were harvested from E14.5 mice and cultured as previously described.^[^ ^64^ ^]^

### Human Samples

Serum (*n* = 8) and feces (*n* = 9) samples from AML patients or healthy volunteers were collected from the Affiliated Jinhua Hospital of Zhejiang University School of Medicine between January 2024 and May 2024. The study had been approved by the Affiliated Jinhua Hospital of Zhejiang University School of Medicine (2024‐Ethical Review‐92). Informed consent was obtained prior to the acquisition of samples from the human participants.

### Mice

*Ubc^ERT2Cre/+^*, and *Villin^Cre/+^* mice were obtained from Shanghai Model Organisms Center, Inc. (Shanghai, China). C57BL/6 (B6) (CD45.2) mice were obtained from Cavens Laboratory Animal Co., Ltd. (Changzhou, China). Ly5.1 (CD45.1) mice were kindly provided by Professor Hui Wang (Xuzhou, China). p53 knock‐out mice were obtained from Jackson Laboratory (Ellsworth, Maine, US). *Rosa26^mSTAT3/mSTAT3^* knock‐in mice were generated as previously described,^[^ ^21^ ^]^ and crossed with *Ubc^ERT2Cre/+^* mice or *Villin^Cre/+^* mice to obtain *Rosa26^mSTAT3/mSTAT3^;Ubc^ERT2Cre/+^* mice or *Rosa26^mSTAT3/mSTAT3^;Villin^Cre/+^* mice, respectively. To obtain *Rosa26^mSTAT3/mSTAT3^;Ubc^ERT2Cre/+^;P53^+/−^* mice, *Rosa26^mSTAT3/mSTAT3^;Ubc^ERT2Cre/+^* mice were crossed with *P53^+/−^* mice. Mice were strictly bred and maintained under protocols approved by the Institutional Animal Care and Use Committee at Xuzhou Medical University (Approval No. 202104A077).

### Mitochondrial STAT3 Induction

To induce mitochondrial STAT3 expression, mitochondrial STAT3 knock‐in mice were given 75 mg kg^−1^ 4‐hydroxytamoxifen (4‐OHT, HY‐16950, MedChemExpress, USA) solution every other day, 7 times by i.p. injection.

### Antibiotic Treatment

Briefly, an antibiotic cocktail containing penicillin (1 g L^−1^, G768670, Macklin, Shanghai, China), neomycin (1 g L^−1^, HY‐B0470, MedChemExpress, USA), metronidazole (1 g L^−1^, M813526, Macklin, Shanghai, China), and vancomycin (0.25 g L^−1^, HY‐B6071, MedChemExpress, USA) was prepared and applied in drinking water. Mice were maintained with normal water or ABX‐supplemented water for 21 days as previously described.^[^ ^65^ ^]^

### Total Body Radiation

The mice were exposed to 7.5 or 5 Gy irradiation by using a GSR C1 137 cesium gamma irradiator (Gamma‐Service Medical, Bautzner, Germany). After radiation, mice were housed in SPF or dirty conditions and provided standard chow and water unless otherwise noted. The survival and/or the changes in body weight and other body parameters after radiation were examined over 30 days (SPF condition) or 7 days (nonsterile condition). A clinical score was determined using a cumulative scoring system based on weight loss, physical appearance, posture, mobility, and food consumption. More details are listed in Table S2 (Supporting Information).

*In* in vitro experiments, mouse BM cells or fetal livers were treated as indicated and followed by 5 Gy irradiation.

### Probiotic Strain Culture

*L. johnsonii, B. pseudolongum, and F. rodentium* were purchased from BeNa Culture Collection (strain numbers: BNCC135265, BNCC135158, and BNCC363015, respectively).

*L. johnsonii* was cultured in MRS broth (Solarbio, Beijing, China). *B. pseudolongum* was cultured in MRS broth with 0.05% L‐cysteine (Aladdin, Shanghai, China). *F. rodentium* was cultured in PYG Medium (modified) (TOPBIO, Qingdao, China) with 0.0005% hematin chloride solution, 0.0001% vitamin K1, and 0.005% L‐cysteine hydrochloride. *L. johnsonii*, *B. pseudolongum*, and *F. rodentium* were grown in an anaerobic chamber (DG250 Anaerobic Workstation, Do Whitely Scientific, UK). Bacterial supernatant (SUP) was derived from cultures of the indicated strains and filtered through 0.22 µm filters.

### Probiotic Administration

SPF C57BL/6J mice were treated with streptomycin (18 mg per mouse, HY‐B1906, MedChemExpress, USA) by oral gavage 1 day, and followed by the indicated treatment (the same volume of bacteria culture medium, 1.5 × 10^7^ CFU/150 µL *F. rodentium*, 2×10^8^ CFU/150 µL *L. Johnsonii*, or 5 × 10^8^ CFU/150 µL *B. pseudolongum*) by oral gavage. After the last administration, mice were given 7.5 or 5 Gy total body radiation and received the same amounts of the indicated probiotics strain every other day.

### Butyrate Treatment

For butyrate diet experiments, mice were fed either normal chow or food containing 5% butyrate (Jiangsu Synergy Pharmaceutical and Biological Company) 2 weeks before irradiation.

SPF C57BL/6J mice received streptomycin treatment (18 mg per mouse) by oral gavage and followed by the same volume of PBS or 20 mg kg^−1^ butyrate (HY‐B0350A, MedChemExpress, USA) treatment for 5 days. After the last treatment, mice were given 7.5 or 5 Gy total body radiation and received PBS or butyrate every other day.

### Bone Marrow Transplantation Assay

For the competitive reconstitution assay, total BM cells (CD45.2, 5×10^6^) from *Rosa26^mSTAT3/mSTAT3^* or *Rosa26^mSTAT3/mSTAT3^;Villin^Cre/+^* mice were transplanted into lethally irradiated Ly5.1 (CD45.1) recipients together with competitor BM cells (5×10^6^) from Ly5.1 mice. One month later, peripheral blood (PB) was collected by orbital plexus bleeding and stained with PE‐anti‐CD45.1 (#110707, 1:200, Biolegend) and FITC‐anti‐CD45.2 (#109805, 1:200, Biolegend) antibodies, and subjected to FACS analysis.

### Blood Cell Counts

Mouse PB samples were obtained by orbital plexus bleeding. White blood cells (WBC), red blood cells (RBC), and platelets of complete blood counts were acquired through the Mindray BC‐5300 Auto Hematology Analyzer. PB samples were stained with FITC‐anti‐CD3 (#100204, 1:200, Biolegend) and APC‐Cy7‐anti‐CD19 (#115530, 1:200, Biolegend) antibodies, and subjected to FACS analysis to assess the number of T lymphocytes and B lymphocytes. 123count eBeads™ Counting Beads (01‐1234‐42, Invitrogen) were added to each sample.

### Flow Cytometry Analysis

Briefly, single‐cell suspensions were obtained from mouse BM. RBCs were lysed with red cell lysis buffer. After incubation with an anti‐CD16/32 antibody (2.4G2, BD Biosciences) to block the nonspecific antibody binding to the cells. The samples were stained with FITC‐anti‐Lineage (e.g., CD3, CD19 (#115506, 1:200, Biolegend), CD11b (#101206, 1:200, Biolegend), Ly6G (#127605, 1:200, Biolegend)), PE‐Cy5‐anti‐c‐Kit (#105810, 1:200, Biolegend), PB‐anti‐Sca‐1 (#108120, 1:200, Biolegend), PE‐anti‐CD34 (#55 138, 1:200, BD Biosciences), PE‐Cy7‐anti‐CD16 (#101318, 1:200, Biolegend)) and APC‐anti‐CD135 (#135 310, 1:200, Biolegend) antibodies at 4 °C for 20 min, and subjected to FACS for LT‐LSK, ST‐LSK, MPPs, hemapoietic progenitor cells (HPCs), GMPs, CMPs, and MEPs analysis.

### Cell Isolation and Culture

Single‐cell suspensions were obtained from mouse BM, and red cells of the single‐cell suspensions were lysed with red cell lysis buffer. The cells were stained with the FITC‐anti‐Lineage, PE‐Cy5‐anti‐c‐Kit, APC‐anti‐Sca‐1 (#1081112, 1:200, Biolegend), BV605‐anti‐CD150 (#115927, 1:200, Biolegend) and PB‐anti‐CD48 (#103417, 1:200, Biolegend) antibodies. Lin^−^Sca‐1^+^c‐Kit^+^CD48^−^CD150^+^ cells were sorted by flow cytometry and cultured with Stem Span medium (Stem Cell).

The c‐Kit^+^ cells were isolated by c‐Kit microbeads (Miltenyi Biotec Kit) and then sorted by flow cytometry.

### Short Hairpin (sh) RNA and Transfection

The control siRNA and siPKM2 (5′‐GUGGAGGCCUCUUAUAAGUTT‐3′, 5′‐ACUUAUAAGAGGCCUCCACTT‐3′) were purchased from Jima (Shanghai, China). Cells were transfected with the indicated siRNAs.

### Plasmid DNA, Lentivirus, and Infection

Nuclear‐PKM2 cDNA was amplified and sub‐cloned into the pLVX‐TetOne‐Puro vector. The pLVX‐TetOne‐Puro vector was a gift from Professor Feng Guo (Xuzhou Medical University, Xuzhou, China). Lentivirus was generated. Briefly, 293T cells were cotransfected with pMD2G, psPAX2, and nuclear‐PKM2 plasmid DNA. After 48 h, the culture medium was harvested and centrifuged. The fetal liver cells were infected with the nuclear‐PKM2 lentivirus in the presence of 10 µg mL^−1^ polybrene. After 24 h, the culture medium was removed, and StemSpan SFEM containing 10 ng mL^−1^ SCF (#250‐03, PeproTech) and 10 ng mL^−1^ FLT3L (#250‐31L, PeproTech), 200 ng mL^−1^ puromycin, and 300 ng mL^−1^ DOX (HY‐N0565, MedChemExpress, USA) was added to the wells. Seven days later, the infected cells were harvested and subjected to the indicated experiments.

### Hematopoietic Colony‐Forming Assays

BM cells from every two mice were pooled into one sample, and Lin^−^Sca‐1^+^c‐Kit^+^CD48^−^CD150^+^ cells (purified BM LSK cells) were sorted. Then, 10 000 of the sorted purified BM LSK cells were cultured in Methocult GF M3434 in the presence or absence of butyrate (25 µm) for 7–10 days to perform the methylcellulose‐based colony‐forming unit (CFU) assays.

### Metabolic Assays

The extracellular acidification rate (ECAR) was measured with an XF 24 extracellular flux analyzer (Seahorse Bioscience). Briefly, 1.5×10^5^ c‐Kit^+^ cells from *Rosa26^mSTAT3/mSTAT3^* mice or *Rosa26^mSTAT3/mSTAT3^*;*Villin^Cre/+^* mice treated with 7.5 Gy irradiation were seeded in each well of a Seahorse XF 24 plate precoated with Corningcell‐Tak (354240, Corning, USA) in the presence or absence of 5 mm butyrate and incubated overnight. The following day, the cells were preincubated at 37 °C for a minimum of 45 min in the absence of CO_2_ in RPMI (Seahorse, Agilent) with 25 mm glucose (Vicemed, Xuzhou, China) and 1 mm pyruvate (Vicemed) with the pH adjusted to 7.4. The ECAR was measured with the following reagents: 1 µm oligomycin (HY‐N6782, MedChemExpress, USA), 100 mm 2‐DG (HY‐13966, MedChemExpress, USA), and 30 mm glucose. The results were analyzed with Wave software version 2.4.0 (Agilent), and ECAR measurements were normalized to the cell number.

### In Silico Analysis for Clinical Data

For the analysis of *Holdemanella biformis* abundances in ALL patients, data were extracted from the GMrepo database (https://gmrepo.humangut.info).

### Fecal DNA Extraction and Quantification

Fecal DNA was extracted using the CWBIO Stool Genomic DNA Kit (CWBIO, China) according to the manufacturer's protocol, and the concentration was measured by Nanodrop Lite (Thermo). Quantitative PCR assays were performed using LightCycler 480 SYBR Green Master Mix (Roche (USA) 04887352001, Roche, USA). The primers used for PCR are listed in Table S3 (Supporting Information).

### Fecal 16S rRNA Microbial Analysis and Metagenomics Sequencing

Fresh feces collected from individual mice were stored at −80 °C until analysis. For 16S rRNA microbial analysis, fecal DNA extraction and sequencing were performed by Majorbio (Shanghai, China). Data processing was performed.

For metagenomics sequences, fecal DNA extraction and sequencing were performed, and data were obtained. Briefly, 0.5 g of stool was used to extract total genomic DNA with the PF Mag‐Bind Stool DNA Kit (Omega Biotek, Norcross, GA, U.S.) according to the manufacturer's instructions. The DNA extract was fragmented to an average size of ≈400 bp using Covaris M220 (Gene Company Limited, China) for paired‐end library construction. A paired‐end library was constructed using NEXTFLEX Rapid DNA‐Seq (Bioo Scientific, Austin, TX, USA). Paired‐end sequencing was performed on an Illumina NovaSeq 6000 (Illumina Inc., San Diego, CA, USA) by Majorbio Bio‐Pharm Technology Co., Ltd. (Shanghai, China) using a NovaSeq 6000 S4 Reagent Kit according to the manufacturer's instructions (www.illumina.com). The data were analyzed using the Majorbio Cloud Platform (www.majorbio.com).

### Measurement of SCFAs in Probiotic Culture Medium, Feces, or Serum

The culture medium was harvested, centrifuged, filtered, and subjected to gas chromatography‐mass spectrometry (GC‐MS) for SCFA analysis. At the end of the mouse experiments, the feces were collected and subjected to GC–MS analysis to analyze SCFAs. The serum of AML patients was collected and subjected to GC‐MS analysis to analyze butyrate concentration.

### Histological Analysis and Damage Scores

Tissues were fixed overnight in 10% formalin. The formalin‐fixed tissues were paraffin‐embedded at the Histopathology Core Facility. Sections (5 µm) were cut and stained with hematoxylin and eosin (H&E). Intestinal epithelial injury was classified using the Chiu's method: 0, normal intestinal mucosal villi;^[^ ^66^ ^]^ 1, capillary hyperemia and cystic gaps under the epithelium at the villus apex; 2, cystic gaps enlarged under the epithelium, edema extended in the lamina propria, and central cheliferous vessels dilated; 3, degeneration and necrosis of IECs, severe edema in the lamina propria, and rarely seen abscission in villus apexes; 4, degeneration, necrosis, and exfoliation of IECs, hyperemia, dilation of capillary, uncovering of lamina propria, abscission in some villi, and 5, bleeding, ulceration, disintegration of the lamina propria, and abscission of villi.^[^ ^67^ ^]^

### Multiplexed Immunofluorescence Staining and Confocal Microscopy

The fixed BM sections were stained with anti‐PKM2 (#15822‐1‐AP,1:120), anti‐Ly6G (#A22270,1:500), anti‐CD19 (#90176S,1:2000), and anti‐c‐Kit (#18696‐1‐AP,1:200) antibodies using a PANO 7‐plex IHC kit (PANO, Beijing, China). The infected fetal liver cells were stained with anti‐PKM2 antibody using a PANO 7‐plex IHC kit (PANO, Beijing, China). Images were obtained under a Leica STELLARIS 5 confocal microscope (Leica, Germany).

### TUNEL Assay

Apoptotic cells were examined using the One Step TUNEL Apoptosis Detection Kit (Cyanine 3) (#HY‐K1079, MedChemExpress) according to the manufacturer's protocol. Images were obtained under a Leica STELLARIS 5 confocal microscope.

### ROS Assay

The IECs were harvested and stained with 10 µm H2DCFDA (#HY‐D0940, MedChemExpress) in PBS for 20 min at 37 °C. The cells were washed with PBS and resuspended in PBS. The fluorescence intensity of H2DCFA was detected by FACS and analyzed in FlowJo software.

### Immunoblot Assay

Briefly, c‐Kit^+^ cells were isolated by c‐Kit microbeads plus flow cytometry, and cell lysates were extracted and separated on 8% or 4–12% SDS‒PAGE gels. After semi‐dry transfer, the membranes were sequentially probed with the indicated antibodies. Anti‐p53 (1C12) (cat# 2524, 1:1000), anti‐STAT1 (D1K9Y) (cat#14994S, 1:2000), anti‐STAT2 (D9J7L) (cat#72604S, 1:2000), anti‐STAT3 (D3Z2G) (cat#12640, 1:2000), anti‐ERK (137F5) (cat#4695S, 1:2000), anti‐p‐ERK (D13.14.4E)(cat# 4370S, 1:1000) and anti‐VDAC (D73D12) (cat#4661S, 1:2000) antibodies were purchased from Cell Signaling Technology. Anti‐HK1 (cat#19662‐1‐AP, 1:2000), anti‐HK2 (cat#22029‐1‐AP, 1:2000), anti‐PKM1 (cat#15821‐1‐AP, 1:2000), anti‐PKM2 (cat#15822‐1‐AP, 1:2000), anti‐ZO‐1 (cat#21773‐1‐AP, 1:2500), anti‐Occludin (cat#27260‐1‐AP, 1:2000) anti‐GAPDH (cat#60004‐1‐Ig, 1:2000) and anti‐β‐actin (cat#66009‐1‐Ig, 1:2000) antibodies were purchased from ProteinTech. Anti‐Lysozyme (cat#ET1609‐35, 1:1000) and anti‐BAX (cat#ET1603‐34, 1:2000) antibodies were purchased from HUABIO (Hangzhou, China). Anti‐P‐PKM2 (ser37) (cat#AF7231.1:1500) antibody was purchased from Affinity (Changzhou, China). Anti‐MMP7 (cat#WL04679, 1:1000) antibody was purchased from Wanleibio (Shenyang, China).

### Real‐Time RT‒PCR

Total RNA was isolated using the Cell‐to‐CT 1‐step Power SYBR Green kit (Invitrogen) and subjected to real‐time RT‒PCR using SYBR Green I Master (Roche Diagnostics GmbH) on a LightCycler 480 system (Roche). The primers used for real‐time RT‒PCR can be found in Table S4 (Supporting Information).

### Statistical Analysis

Statistical analysis was performed to assess the difference using an unpaired Student's t‐test, one‐way ANOVA, followed by Tukey's multiple comparisons test to minimize Type I errors by Prism statistical analysis software (GraphPad Software, San Diego, CA). Spearman's correlation analysis was performed to analyze the correlation between F. rodentium abundance and butyrate kinase expression. The log‐rank (Mantel‐Cox) test and the Gehan‐Breslow‐Wilcoxon test were used to compare the differences in survival rates between groups. Except for the indicated experiment, other experiments were repeated twice. Data are presented as the mean ± SEM. Significance is indicated as follows: ^**^p < 0.01, ^*^p < 0.05.

### Ethics Approval Statement

The study has been approved by the Affiliated Jinhua Hospital of Zhejiang University School of Medicine (2024‐Ethical Review‐92) and the Institutional Animal Care and Use Committee at Xuzhou Medical University (Approval No. 202104A077)

## Conflict of Interest

J.Y., H.Z., and Y.P. filed a patent on the application of F. rodentium and its metabolites in HSC protection. The other authors declare that no competing interests exist.

## Author Contributions

H.Z., H.G., N.S., and R.X. contributed equally to the work. J.Y. did conception and design. H.Z., H.G., N.S., Y.P., and J.Y. did development of methodology. H.Z., F.D., X.C., and J.Y. did acquisition of data (provided animals, acquired and managed patients, provided facilities, etc.). H.Z., N.S., J.Z., C.Y., B.J., R.J., X.W., S.G., Y.Q., X.L., R.L., and J. Y. did analysis and interpretation of data (e.g., statistical analysis, biostatistics, and computational analysis). H.Z., N.S., Y.P., T.I., and J.Y. did writing, review, and/or revision of the manuscript. Y.P. and J.Y. did administrative, technical, or material support (i.e., reporting or organizing Data and constructing databases). J.Y. did study supervision.

## Supporting information



Supporting Information

## Data Availability

The data that support the findings of this study are available from the corresponding author upon reasonable request.

## References

[1] M. L. Balmer , C. M. Schürch , Y. Saito , M. B. Geuking , H. Li , M. Cuenca , L. V. Kovtonyuk , K. D. McCoy , S. Hapfelmeier , A. F. Ochsenbein , M. G. Manz , E. Slack , A. J. Macpherson , J. Immunol. 2014, 193, 5273. 25305320 10.4049/jimmunol.1400762

[2] S. Lee , H. Kim , G. You , Y.‐M. Kim , S. Lee , V.‐H. Le , O. Kwon , S.‐H. Im , Y.‐M. Kim , K. S. Kim , Y. C. Sung , K. H. Kim , C. D. Surh , Y. Park , S.‐W. Lee , Blood 2019, 134, 1312. 31387916 10.1182/blood.2019000495 PMC6888141

[3] H. S. Deshmukh , Y. Liu , O. R. Menkiti , J. Mei , N. Dai , C. E. O'Leary , P. M. Oliver , J. K. Kolls , J. N. Weiser , G. S. Worthen , Nat. Med. 2014, 20, 524. 24747744 10.1038/nm.3542 PMC4016187

[4] D. Zhang , P. S. Frenette , Blood 2019, 133, 2168. 30898860 10.1182/blood-2018-11-844555 PMC6524562

[5] A. Khosravi , A. Yáñez , J. G. Price , A. Chow , M. Merad , H. S. Goodridge , S. K. Mazmanian , Cell Host Microbe 2014, 15, 374. 24629343 10.1016/j.chom.2014.02.006 PMC4144825

[6] D. Zhang , G. Chen , D. Manwani , A. Mortha , C. Xu , J. J. Faith , R. D. Burk , Y. Kunisaki , J.‐E. Jang , C. Scheiermann , M. Merad , P. S. Frenette , Nature 2015, 525, 528. 26374999 10.1038/nature15367 PMC4712631

[7] A. Staffas , M. Burgos da Silva , A. E. Slingerland , A. Lazrak , C. J. Bare , C. D. Holman , M. D. Docampo , Y. Shono , B. Durham , A. J. Pickard , J. R. Cross , C. Stein‐Thoeringer , E. Velardi , J. J. Tsai , L. Jahn , H. Jay , S. Lieberman , O. M. Smith , E. G. Pamer , J. U. Peled , D. E. Cohen , R. R. Jenq , M. R. M. van den Brink , Cell Host Microbe 2018, 23, 447. 29576480 10.1016/j.chom.2018.03.002 PMC5897172

[8] K. S. Josefsdottir , M. T. Baldridge , C. S. Kadmon , K. Y. King , Blood 2017, 129, 729. 27879260 10.1182/blood-2016-03-708594 PMC5301822

[9] D. Zhang , X. Gao , H. Li , D. K. Borger , Q. Wei , E. Yang , C. Xu , S. Pinho , P. S. Frenette , Cell Stem Cell 2022, 29, 232. 35065706 10.1016/j.stem.2021.12.009 PMC8818037

[10] H. Guo , W.‐C. Chou , Y. Lai , K. Liang , J. W. Tam , W. J. Brickey , L. Chen , N. D. Montgomery , X. Li , L. M. Bohannon , A. D. Sung , N. J. Chao , J. U. Peled , A. L. C. Gomes , M. R. M. van den Brink , M. J. French , A. N. Macintyre , G. D. Sempowski , X. Tan , R. B. Sartor , K. Lu , J. P. Y. Ting , Science 2020,370, aay9097. 10.1126/science.aay9097 PMC7898465 33122357

[11] N. Vannini , V. Campos , M. Girotra , V. Trachsel , S. Rojas‐Sutterlin , J. Tratwal , S. Ragusa , E. Stefanidis , D. Ryu , P. Y. Rainer , G. Nikitin , S. Giger , T. Y. Li , A. Semilietof , A. Oggier , Y. Yersin , L. Tauzin , E. Pirinen , W.‐C. Cheng , J. Ratajczak , C. Canto , M. Ehrbar , F. Sizzano , T. V. Petrova , D. Vanhecke , L. Zhang , P. Romero , A. Nahimana , S. Cherix , M. A. Duchosal , et al., Cell Stem Cell 2019, 24, 405. 30849366 10.1016/j.stem.2019.02.012

[12] W. Li , X. Wang , Y. Dong , Q. Huo , T. Yue , X. Wu , L.u Lu , J. Zhang , Y.u Zhao , H. Dong , D. Li , Aging Cell 2023, 22, 13976. 10.1111/acel.13976 PMC10652312 37650560

[13] I. Rowland , G. Gibson , A. Heinken , K. Scott , J. Swann , I. Thiele , K. Tuohy , Eur. J. Nutr. 2018, 57, 1. 10.1007/s00394-017-1445-8 PMC5847071 28393285

[14] Q. Zhang , M. Raoof , Y.u Chen , Y. Sumi , T. Sursal , W. Junger , K. Brohi , K. Itagaki , C. J. Hauser , Nature 2010, 464, 104. 20203610 10.1038/nature08780 PMC2843437

[15] Y. Zhang , J. Zhang , L. Duan , Pharmacol. Res. 2022, 186, 106530. 36349593 10.1016/j.phrs.2022.106530

[16] H. Fujiwara , K. Seike , M. D. Brooks , A. V. Mathew , I. Kovalenko , A. Pal , H.o‐J. Lee , D. Peltier , S. Kim , C. Liu , K. Oravecz‐Wilson , L.u Li , Y. Sun , J. Byun , Y. Maeda , M. S. Wicha , T. L. Saunders , A. Rehemtulla , C. A. Lyssiotis , S. Pennathur , P. Reddy , Nat. Immunol. 2021, 22, 1440. 34686860 10.1038/s41590-021-01048-3 PMC9351914

[17] J. Ma , C. Coarfa , X. Qin , P. E. Bonnen , A. Milosavljevic , J. Versalovic , K. Aagaard , BMC Genomics 2014, 15, 257. 24694284 10.1186/1471-2164-15-257 PMC4234434

[18] Y.i Wang , H. Lai , T. Zhang , J. Wu , H. Tang , X. Liang , D. Ren , J. Huang , W. Li , Neurosci. Biobehav. Rev. 2023, 153, 105403. 37742989 10.1016/j.neubiorev.2023.105403

[19] G. Hua , T. H. Thin , R. Feldman , A. Haimovitz‐Friedman , H. Clevers , Z. Fuks , R. Kolesnick , Gastroenterology 2012, 143, 1266. 22841781 10.1053/j.gastro.2012.07.106 PMC3480544

[20] Y. Li , R. Tinoco , L. Elmén , I. Segota , Y. Xian , Y.u Fujita , A. Sahu , R. Zarecki , K. Marie , Y. Feng , A. Khateb , D. T. Frederick , S. K. Ashkenazi , H. Kim , E. G. Perez , C.‐P. Day , R. S. Segura Muñoz , R. Schmaltz , S. Yooseph , M. A. Tam , T. Zhang , E. Avitan‐Hersh , L. Tzur , S. Roizman , I. Boyango , G. Bar‐Sela , A. Orian , R. J. Kaufman , M. Bosenberg , C. R. Goding , et al., Nat. Commun. 2019, 10, 1492. 30940817 10.1038/s41467-019-09525-y PMC6445090

[21] R. Li , X. Li , J. Zhao , F. Meng , C. Yao , E. Bao , N.a Sun , X. Chen , W. Cheng , H. Hua , X. Li , B.o Wang , H. Wang , X. Pan , H. You , J. Yang , T. Ikezoe , Theranostics 2022, 12, 976. 34976224 10.7150/thno.63751 PMC8692896

[22] J. Y. Lee , R. M. Tsolis , A. J. Bäumler , Science 2022, 377, abp9960. 10.1126/science.abp9960 35771903

[23] R. Wang , X. Yang , J. Liu , F. Zhong , C. Zhang , Y. Chen , T. Sun , C. Ji , D. Ma , Nat. Commun. 2022, 13, 2522. 35534496 10.1038/s41467-022-30240-8 PMC9085760

[24] R. E. Vandenbroucke , I. Vanlaere , F. Van Hauwermeiren , E. Van Wonterghem , C. Wilson , C. Libert , Mucosal Immunol. 2014, 7, 579. 24129163 10.1038/mi.2013.76

[25] M. Heidarian , I. J. Jensen , S. K. Kannan , L. L. Pewe , M. Hassert , S. Park , H.‐H. Xue , J. T. Harty , V. P. Badovinac , Proc. Natl. Acad. Sci. U S A 2023, 120, 2302785120. 10.1073/pnas.2302785120 PMC10318958 37364124

[26] K. Miyamoto , K. Y. Araki , K. Naka , F. Arai , K. Takubo , S. Yamazaki , S. Matsuoka , T. Miyamoto , K. Ito , M. Ohmura , C. Chen , K. Hosokawa , H. Nakauchi , K. Nakayama , K. I. Nakayama , M. Harada , N. Motoyama , T. Suda , A. Hirao , Cell Stem Cell 2007, 1, 101. 18371339 10.1016/j.stem.2007.02.001

[27] A. Krysztofiak , K. Szymonowicz , J. Hlouschek , K. Xiang , C. Waterkamp , S. Larafa , I. Goetting , S. Vega‐Rubin‐de‐Celis , C. Theiss , V. Matschke , D. Hoffmann , V. Jendrossek , J. Matschke , iscience 2021,24, 103366. 34825138 10.1016/j.isci.2021.103366 PMC8603201

[28] O. Witt , K. Sand , A. Pekrun , Blood 2000, 95, 2391. 10733512

[29] W. Yang , Y. Zheng , Y. Xia , H. Ji , X. Chen , F. Guo , C. A. Lyssiotis , K. Aldape , L. C. Cantley , Z. Lu , Nat. Cell Biol. 2012, 14, 1295. 23178880 10.1038/ncb2629 PMC3511602

[30] A. Hamabe , M. Konno , N. Tanuma , H. Shima , K. Tsunekuni , K. Kawamoto , N. Nishida , J. Koseki , K. Mimori , N. Gotoh , H. Yamamoto , Y. Doki , M. Mori , H. Ishii , Proc. Natl. Acad. Sci. USA 2014, 111, 15526. 25313085 10.1073/pnas.1407717111 PMC4217454

[31] M. Payen , I. Nicolis , M. Robin , D. Michonneau , J. Delannoye , C. Mayeur , N. Kapel , B. Berçot , M.‐J. Butel , J. Le Goff , G. Socié , C. Rousseau , Blood Adv. 2020, 4, 1824. 32353108 10.1182/bloodadvances.2020001531 PMC7218439

[32] J. U. Peled , A. L. C. Gomes , S. M. Devlin , E. R. Littmann , Y. Taur , A. D. Sung , D. Weber , D. Hashimoto , A. E. Slingerland , J. B. Slingerland , M. Maloy , A. G. Clurman , C. K. Stein‐Thoeringer , K. A. Markey , M. D. Docampo , M. Burgos da Silva , N. Khan , A. Gessner , J. A. Messina , K. Romero , M. V. Lew , A. Bush , L. Bohannon , D. G. Brereton , E. Fontana , L. A. Amoretti , R. J. Wright , G. K. Armijo , Y. Shono , M. Sanchez‐Escamilla , et al., N. Engl. J. Med. 2020, 382, 822. 32101664 10.1056/NEJMoa1900623 PMC7534690

[33] J. U. Peled , S. M. Devlin , A. Staffas , M. Lumish , R. Khanin , E. R. Littmann , L. Ling , S. Kosuri , M. Maloy , J. B. Slingerland , K. F. Ahr , K. A. Porosnicu Rodriguez , Y. Shono , A. E. Slingerland , M. D. Docampo , A. D. Sung , D. Weber , A. M. Alousi , B. Gyurkocza , D. M. Ponce , J. N. Barker , M.‐A. Perales , S. A. Giralt , Y. Taur , E. G. Pamer , R. R. Jenq , M. R. M. van den Brink , J. Clin. Oncol. 2017, 35, 1650. 28296584 10.1200/JCO.2016.70.3348 PMC5455763

[34] Y. Taur , R. R. Jenq , M.‐A. Perales , E. R. Littmann , S. Morjaria , L. Ling , D. No , A. Gobourne , A. Viale , P. B. Dahi , D. M. Ponce , J. N. Barker , S. Giralt , M. van den Brink , E. G. Pamer , Blood 2014, 124, 1174. 24939656 10.1182/blood-2014-02-554725 PMC4133489

[35] R. R. Jenq , C. Ubeda , Y. Taur , C. C. Menezes , R. Khanin , J. A. Dudakov , C. Liu , M. L. West , N. V. Singer , M. J. Equinda , A. Gobourne , L. Lipuma , L. F. Young , O. M. Smith , A. Ghosh , A. M. Hanash , J. D. Goldberg , K. Aoyama , B. R. Blazar , E. G. Pamer , M. R. M. van den Brink , J. Exp. Med. 2012, 209, 903. 22547653 10.1084/jem.20112408 PMC3348096

[36] D. N. Fredricks , J. Clin. Invest. 2019, 129, 1808. 31042160 10.1172/JCI125797 PMC6486325

[37] C. K. Stein‐Thoeringer , K. B. Nichols , A. Lazrak , M. D. Docampo , A. E. Slingerland , J. B. Slingerland , A. G. Clurman , G. Armijo , A. L. C. Gomes , Y. Shono , A. Staffas , M. Burgos da Silva , S. M. Devlin , K. A. Markey , D. Bajic , R. Pinedo , A. Tsakmaklis , E. R. Littmann , A. Pastore , Y. Taur , S. Monette , M. E. Arcila , A. J. Pickard , M. Maloy , R. J. Wright , L. A. Amoretti , E. Fontana , D. Pham , M. A. Jamal , D. Weber , et al., Science 2019, 366, 1143. 31780560 10.1126/science.aax3760 PMC7003985

[38] E. Hayase , T. Hayase , M. A. Jamal , T. Miyama , C.‐C. Chang , M. R. Ortega , S. S. Ahmed , J. L. Karmouch , C. A. Sanchez , A. N. Brown , R. K. El‐Himri , I. I. Flores , L. K. McDaniel , D. Pham , T. Halsey , A. C. Frenk , V. A. Chapa , B. E. Heckel , Y. Jin , W.‐B. Tsai , R. Prasad , L. Tan , L. Veillon , N. J. Ajami , J. A. Wargo , J. Galloway‐Peña , S. Shelburne , R. F. Chemaly , L. Davey , R. W. P. Glowacki , et al., Cell 2022, 185, 3705. 36179667 10.1016/j.cell.2022.09.007 PMC9542352

[39] Y. Luo , G.‐L. Chen , N. Hannemann , N. Ipseiz , G. Krönke , T. Bäuerle , L. Munos , S. Wirtz , G. Schett , A. Bozec , Cell Metab. 2015, 22, 886. 26387866 10.1016/j.cmet.2015.08.020

[40] I. Hazan‐Halevy , D. Harris , Z. Liu , J. Liu , P. Li , X. Chen , S. Shanker , A. Ferrajoli , M. J. Keating , Z. Estrov , Blood 2010, 115, 2852. 20154216 10.1182/blood-2009-10-230060 PMC2918366

[41] Y. J. Chung , B. B. Park , Y. J. Kang , T. M. Kim , C. J. Eaves , I. H. Oh , Blood 2006, 108, 1208. 16614239 10.1182/blood-2006-01-010199

[42] C. Mantel , S. Messina‐Graham , A. Moh , S. Cooper , G. Hangoc , X.‐Y. Fu , H. E. Broxmeyer , Blood 2012, 120, 2589. 22665934 10.1182/blood-2012-01-404004 PMC3460681

[43] J. F. Bromberg , M. H. Wrzeszczynska , G. Devgan , Y. Zhao , R. G. Pestell , C. Albanese , J. E. Darnell , Cell 1999, 98, 295. 10458605 10.1016/s0092-8674(00)81959-5

[44] A. T. Soderholm , V. A. Pedicord , Immunology 2019, 158, 267. 31509239 10.1111/imm.13117 PMC6856932

[45] G. Li , J. Lin , X. Gao , H. Su , R. Lin , H. Gao , Z. Feng , H. Wu , B. Feng , K. Zuo , Y. Li , W. Wu , L. Fang , Z. Liu , Gut Microbes 2023, 15, 2257269. 37749885 10.1080/19490976.2023.2257269 PMC10524779

[46] Y.‐G. Zhang , X. Zhu , R. Lu , J. S. Messer , Y. Xia , E. B. Chang , J. Sun , Autophagy 2019, 15, 1935. 30894054 10.1080/15548627.2019.1596485 PMC6844505

[47] J. J. Balic , H. Albargy , K. Luu , F. J. Kirby , W. S. N. Jayasekara , F. Mansell , D. J. Garama , D. De Nardo , N. Baschuk , C. Louis , F. Humphries , K. Fitzgerald , E. Latz , D. J. Gough , A. Mansell , Nat. Commun. 2020, 11, 3816. 32732870 10.1038/s41467-020-17669-5 PMC7393113

[48] N. Shulga , J. G. Pastorino , J. Cell Sci. 2014, 127, 896. 24357718 10.1242/jcs.140764 PMC3924205

[49] W. Yoo , N. G. Shealy , J. K. Zieba , T. P. Torres , M. Baltagulov , J. D. Thomas , C. D. Shelton , A. G. McGovern , N. J. Foegeding , E. E. Olsan , M. X. Byndloss , Cell Host Microbe 2024, 32, 887. 38806059 10.1016/j.chom.2024.05.001 PMC11189616

[50] J. Zhou , M. Li , Q. Chen , X. Li , L. Chen , Z. Dong , W. Zhu , Y. Yang , Z. Liu , Q. Chen , Nat. Commun. 2022, 13, 3432. 35701435 10.1038/s41467-022-31171-0 PMC9198027

[51] L. Hu , X. Yin , Y. Zhang , A. Pang , X. Xie , S. Yang , C. Zhu , Y. Li , B. Zhang , Y. Huang , Y. Tian , M. Wang , W. Cao , S. Chen , Y. Zheng , S. Ma , F. Dong , S. Hao , S. Feng , Y. Ru , H. Cheng , E. Jiang , T. Cheng , Blood 2021, 137, 3339. 33881475 10.1182/blood.2020007362 PMC8233686

[52] S. Deleu , K. Machiels , J. Raes , K. Verbeke , S. Vermeire , Ebiomedicine 2021,66, 103293. 33813134 10.1016/j.ebiom.2021.103293 PMC8047503

[53] E. Zagato , C. Pozzi , A. Bertocchi , T. Schioppa , F. Saccheri , S. Guglietta , B. Fosso , L. Melocchi , G. Nizzoli , J. Troisi , M. Marzano , B. Oresta , I. Spadoni , K. Atarashi , S. Carloni , S. Arioli , G. Fornasa , F. Asnicar , N. Segata , S. Guglielmetti , K. Honda , G. Pesole , W. Vermi , G. Penna , M. Rescigno , Nat. Microbiol. 2020, 5, 511. 31988379 10.1038/s41564-019-0649-5 PMC7048616

[54] K. Pant , A. K. Yadav , P. Gupta , R. Islam , A. Saraya , S. K. Venugopal , Redox Biol. 2017, 12, 340. 28288414 10.1016/j.redox.2017.03.006 PMC5350572

[55] G. Fu , S. Chen , L. Liang , X. Li , P. Tang , X. Rao , M. Pan , X. Xu , Y. Li , Y.e Yao , Y.i Zhou , J. Gao , S. Mo , S. Cai , J. Peng , Z. Zhang , H. Clevers , J. Gao , G. Hua , Cancer Lett. 2021, 501, 20. 33359449 10.1016/j.canlet.2020.12.034

[56] Z. Li , C.‐X. Yi , S. Katiraei , S. Kooijman , E. Zhou , C. K. Chung , Y. Gao , J. K. van den Heuvel , O. C. Meijer , J. F. P. Berbée , M. Heijink , M. Giera , K.o Willems van Dijk , A. K. Groen , P. C. N. Rensen , Y. Wang , Gut 2018, 67, 1269. 29101261 10.1136/gutjnl-2017-314050

[57] A. Rainwater , A. D. Güler , J. Neurosci. Methods 2022, 365, 109384. 34634282 10.1016/j.jneumeth.2021.109384 PMC8608720

[58] H. Duan , J. Hu , Y. Deng , J. Zou , W. Ding , Q. Peng , R. Duan , J. Sun , J. Zhu , Nutrients 2023, 16, 9. 38201839 10.3390/nu16010009 PMC10781073

[59] H. N. Sanchez , J. B. Moroney , H. Gan , T. Shen , J. L. Im , T. Li , J. R. Taylor , H. Zan , P. Casali , Nat. Commun. 2020, 11, 60. 31896754 10.1038/s41467-019-13603-6 PMC6940392

[60] Z. Lyu , G. Yuan , Y. Zhang , F. Zhang , Y. Liu , Y. Li , G. Li , Y. Wang , M. Zhang , Y. Hu , Y. Guo , D. Liu , Microbiome 2024, 12, 215. 39438898 10.1186/s40168-024-01920-y PMC11495078

[61] D. Anastasiou , Y. Yu , W. J. Israelsen , J.‐K. Jiang , M. B. Boxer , B. S. Hong , W. Tempel , S. Dimov , M. Shen , A. Jha , H. Yang , K. R. Mattaini , C. M. Metallo , B. P. Fiske , K. D. Courtney , S. Malstrom , T. M. Khan , C. Kung , A. P. Skoumbourdis , H. Veith , N. Southall , M. J. Walsh , K. R. Brimacombe , W. Leister , S. Y. Lunt , Z. R. Johnson , K. E. Yen , K. Kunii , S. M. Davidson , H. R. Christofk , et al., Nat. Chem. Biol. 2012, 8, 839. 22922757 10.1038/nchembio.1060 PMC3711671

[62] B. Saleme , V. Gurtu , Y. Zhang , A. Kinnaird , A. E. Boukouris , K. Gopal , J. R. Ussher , G. Sutendra , Sci. Transl. Med. 2019, 11, aau8866. 10.1126/scitranslmed.aau8866 30728290

[63] J. Yang , A. W. Jin , J. Han , X. Chen , J. N. Zheng , Y. P. Zhang , Cancer Res. 2021, 81, 898. 33277368 10.1158/0008-5472.CAN-20-0790 PMC8026549

[64] Y. Zhao , J. Zhou , D. Liu , F. Dong , H. Cheng , W. Wang , Y. Pang , Y. Wang , X. Mu , Y. Ni , Z. Li , H. Xu , S. Hao , X. Wang , S. Ma , Q.‐F. Wang , G. Xiao , W. Yuan , B. Liu , T. Cheng , Blood 2015, 126, 2383. 26384355 10.1182/blood-2015-03-633354 PMC4653766

[65] Y. Xu , O. Milburn , T. Beiersdorfer , L. Du , H. Akinbi , D. B. Haslam , Microbiome 2022, 10, 103. 35794664 10.1186/s40168-022-01300-4 PMC9260971

[66] C. J. Chiu , A. H. McArdle , R. Brown , H. J. Scott , F. N. Gurd , Arch. Surg. 1970, 101, 478. 5457245 10.1001/archsurg.1970.01340280030009

[67] K. Mori , H. Morisaki , S. Yajima , T. Suzuki , A. Ishikawa , N. Nakamura , Y. Innami , J. Takeda , Intensive Care Med. 2011, 37, 1849. 21847651 10.1007/s00134-011-2326-x

