# Supplementary material for: Faecalibaculum rodentium Alleviates Ionizing Radiation‐Induced Damage in Mice by Improving Intestinal Integrity and Hematopoiesis via Its Metabolite Butyrate
Source: Adv Sci (Weinh). 2025 Oct 13;13(2):e09383. doi: 10.1002/advs.202509383 (PMC12786293; doi:10.1002/advs.202509383)
Supplement: Supplementary file 1 — Supporting Information [file ADVS-13-e09383-s001.docx]

Supplementary Materials for

***Faecalibaculum rodentium* alleviates ionizing radiation–induced damage in mice by improving intestinal integrity and hematopoiesis via**

**its metabolite butyrate**

Hanyong Zhu *et al.*

*Corresponding author:

Prof. Jing Yang, [jingyang@xzhmu.edu.cn](mailto:jingyang@xzhmu.edu.cn)

Dr. Yuchen Pan, panyuchen@xzhmu.edu

Xin Chen，[jhcx10@foxmail.com](mailto:jhcx10@foxmail.com)

**Supplementary Figures**

**
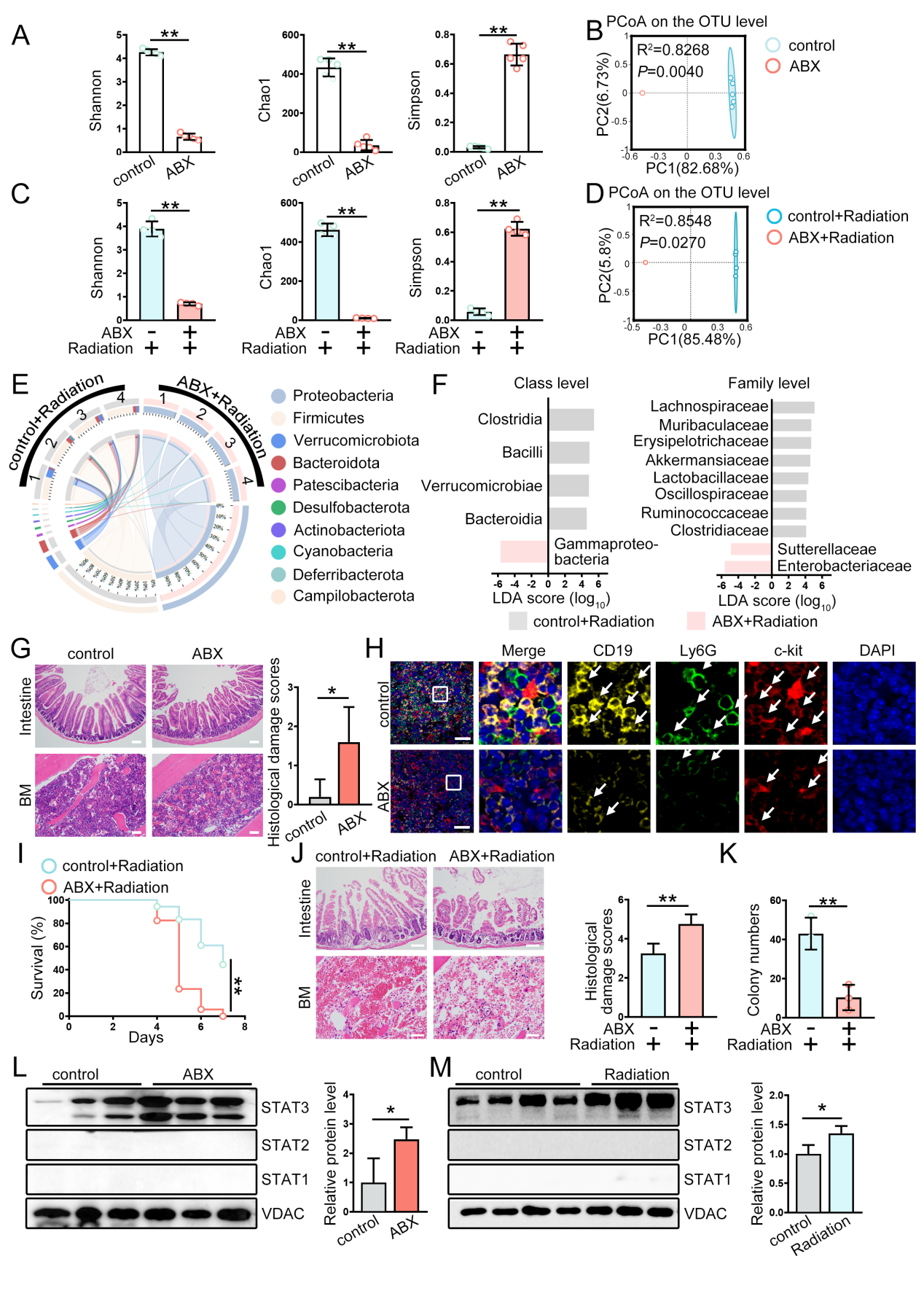
Fig.S1 Gut microbiota dysbiosis increased the sensitivity of mice to irradiation. (A-B)** Before radiation, the mice were treated with normal water or water containing ABX for 21 days. A plot of the Shannon–Wiener diversity index, Chao 1 index, and Simpson index was analyzed. PCoA on the OTU level was analyzed (n = 5 mice/group). **(C-D)** The mice were treated with normal water or ABX water, and followed by radiation. A plot of the Shannon–Wiener diversity index, Chao 1 index, and Simpson index was performed. PCoA on the OTU level was analyzed (n = 4 mice/group). **(E)** Circus plot analysis was performed to characterize the phylum level of each sample (n = 4 mice/group). **(F)** LDA scores were analyzed at class or family levels (n = 4 mice/group). **(A-F)** The figures shown represent a single experiment. **(G)** HE staining of the BM and intestine was performed, and the histological score of the intestine was analyzed. (n = 5 mice/group). **(H)** Repressive IF images showing CD19, Ly6G, and c-Kit expression in bone marrow of control- and ABX-mice before irradiation (n = 5 mice/group). **(G-H)** The figures shown represent one of two independent experiments. **(I)** The survival of irradiated mice was analyzed by Kaplan-Meier analysis (control, n = 18 mice/group; ABX, n = 17 mice/group). The figures show the combination of two experiments. **(J)** Control or ABX-treated mice were irradiated. After 3 days, HE staining was performed, and the histological score of the intestine was quantified (n = 4 mice/group). **(K)** Control or ABX-treated mice were irradiated. 12 h later, c-Kit^+^ cells were sorted, and a colony formation assay was performed. 10-14 days later, the colony number was counted (n = 3 mice/group). **(L-M)** Mitochondrial protein of IECs was extracted, and Western blotting was utilized to examine the indicated protein. VDAC was used as the loading control. The quantification analysis of STAT3 was performed by ImageJ. (n =3 mice/group). **(J-M)** The figures shown represent one of two independent experiments. **P*<0.05, ***P*<0.01.


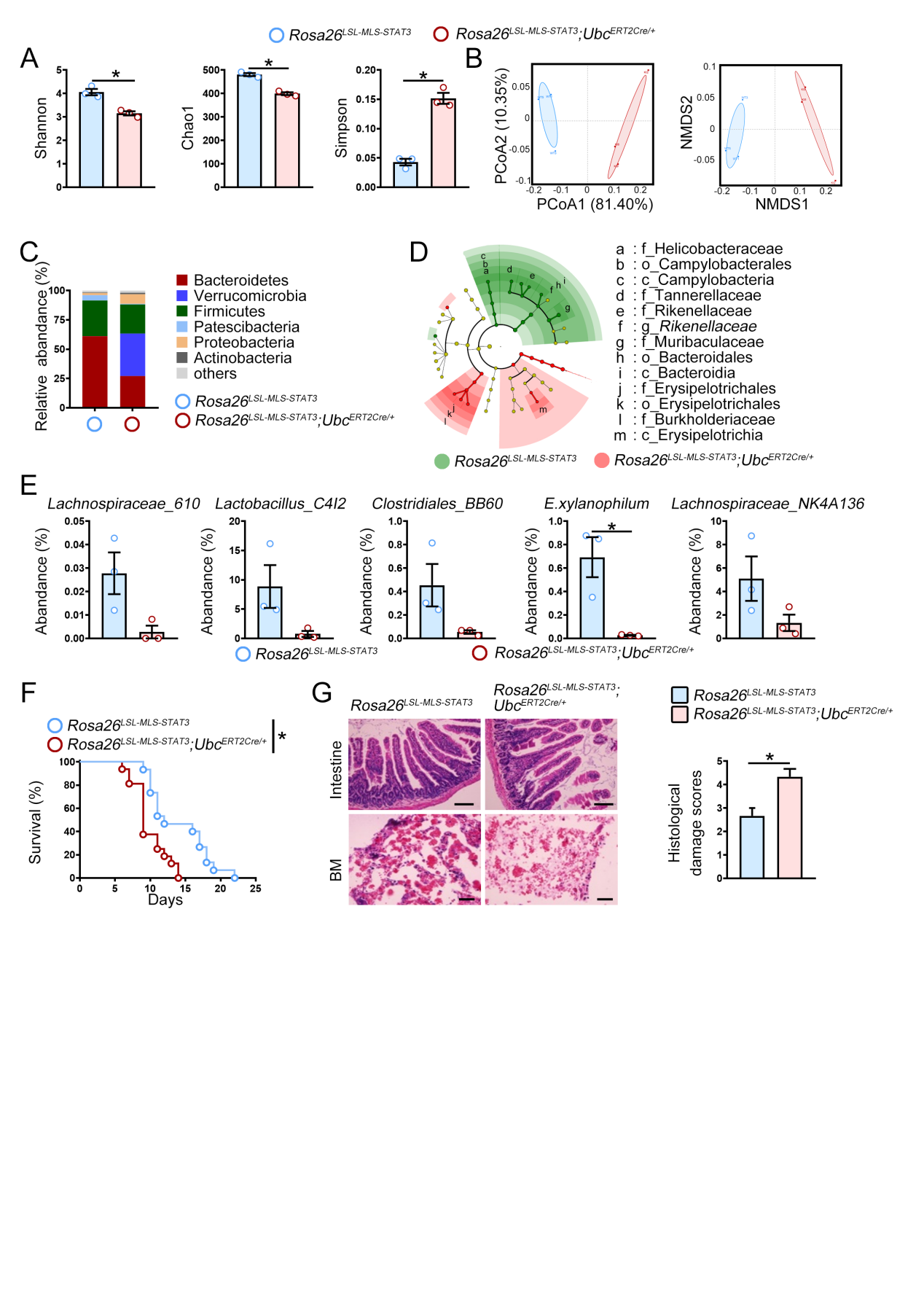


**Fig.S2 *Rosa^26LSL-MLS-mSTAT3^;Ubc^ERT2Cre/+^* mice were sensitive to irradiation.** **(A)** 16S rRNA analysis of the gut microbiota in *Rosa26^LSL-MLS-mSTAT3^* mice and *Rosa26^LSL-MLS-mSTAT3^;Ubc^ERT2Cre/+^* mice after irradiation (n = 3 mice/group). A plot of the Shannon–Wiener diversity index, Simpson index, and Chao 1 index was performed. **(B)** PCoA and NMDS on the OTU level were analyzed. **(C)** The level of the phylum was analyzed. **(D)** A cladogram representation of taxa enriched in *Rosa26^LSL-MLS-mSTAT3^* mice and *Rosa^26LSL-MLS-mSTAT3^;Ubc^ERT2Cre/+^* mice. **(E)** Relative abundance of probiotics at 3 days post-irradiation. **(A-E)** The figures shown represent a single experiment. **(F)** *Rosa26^LSL-MLS-mSTAT3^* (n = 15 mice/group) and *Rosa26^LSL-MLS-mSTAT3^;Ubc^ERT2Cre/+^* mice (n = 16 mice/group) were treated with 4-OHT every other day for 7 times, followed by 7.5 Gy. The survival curves were assessed using a Kaplan-Meier analysis. The figures show the combination of two experiments. **(G)** HE staining of the BM and intestine from irradiated mice was performed, and the histological score of the intestines was quantified. The figures shown represent one of two independent experiments. **P*<0.05.


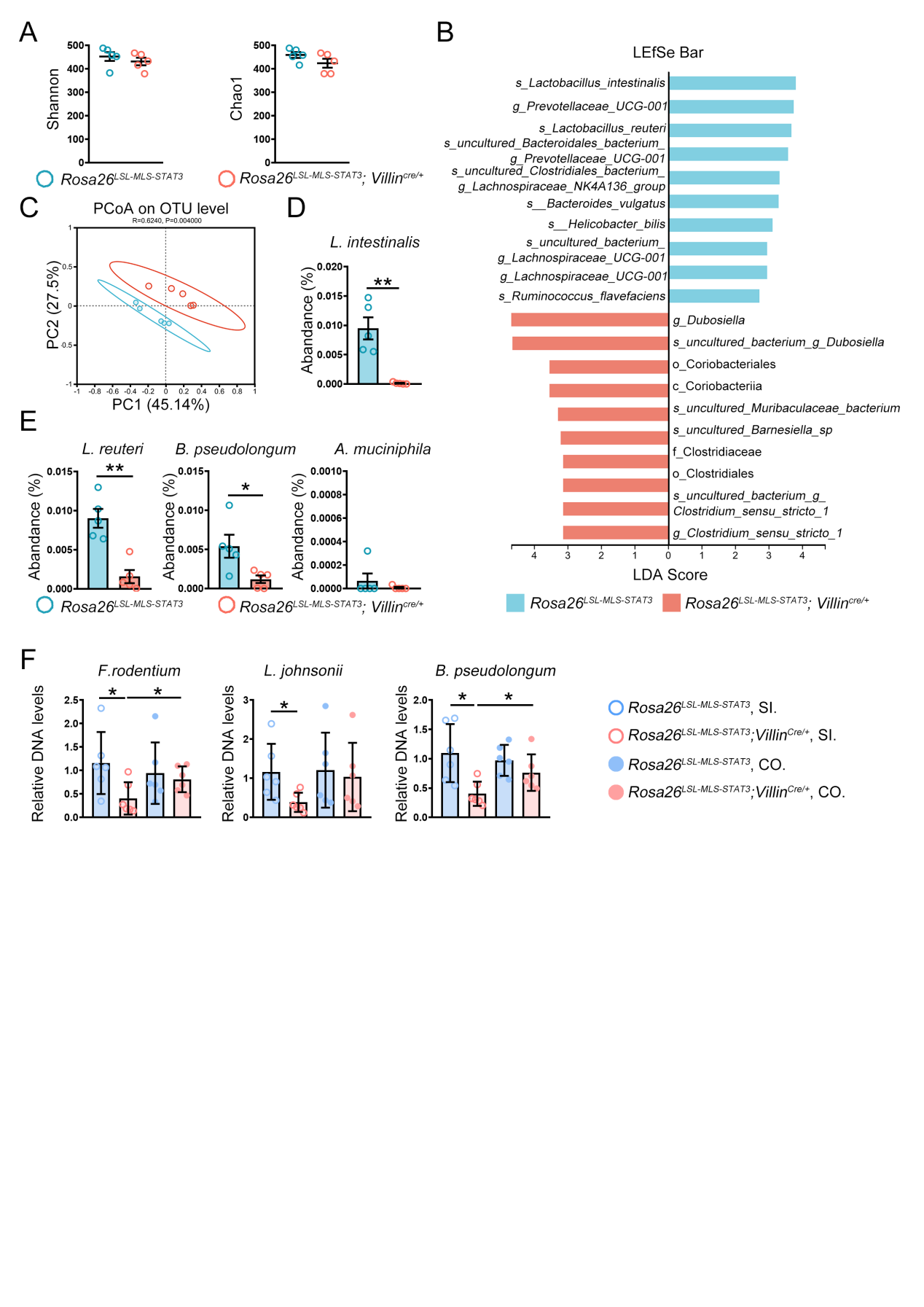


**Fig.S3 Mitochondrial STAT3 altered the gut microbiota. (A)** 16S rRNA analysis of the gut microbiota in *Rosa26^LSL-MLS-mSTAT3^* mice and *Rosa26^LSL-MLS-mSTAT3^;Villin^Cre/+^* mice before irradiation (n = 5 mice/group). A plot of the Shannon–Wiener diversity index and Chao 1 index was performed. **(B)** LDA effect size analysis was performed. **(C)** PCoA analysis score plot was performed. **(D-E)** The relative abundance of probiotics was analyzed on the OTU level. **(A-E)** The figures shown represent a single experiment. **(F)** The relative abundances of *F. rodentium*, *L. johnsonii*, and *B. pseudolongum* in the indicated mice were assessed by real-time PCR (n = 6 mice/group). The figures shown represent one of two independent experiments. **P*<0.05, ***P*<0.01.

**
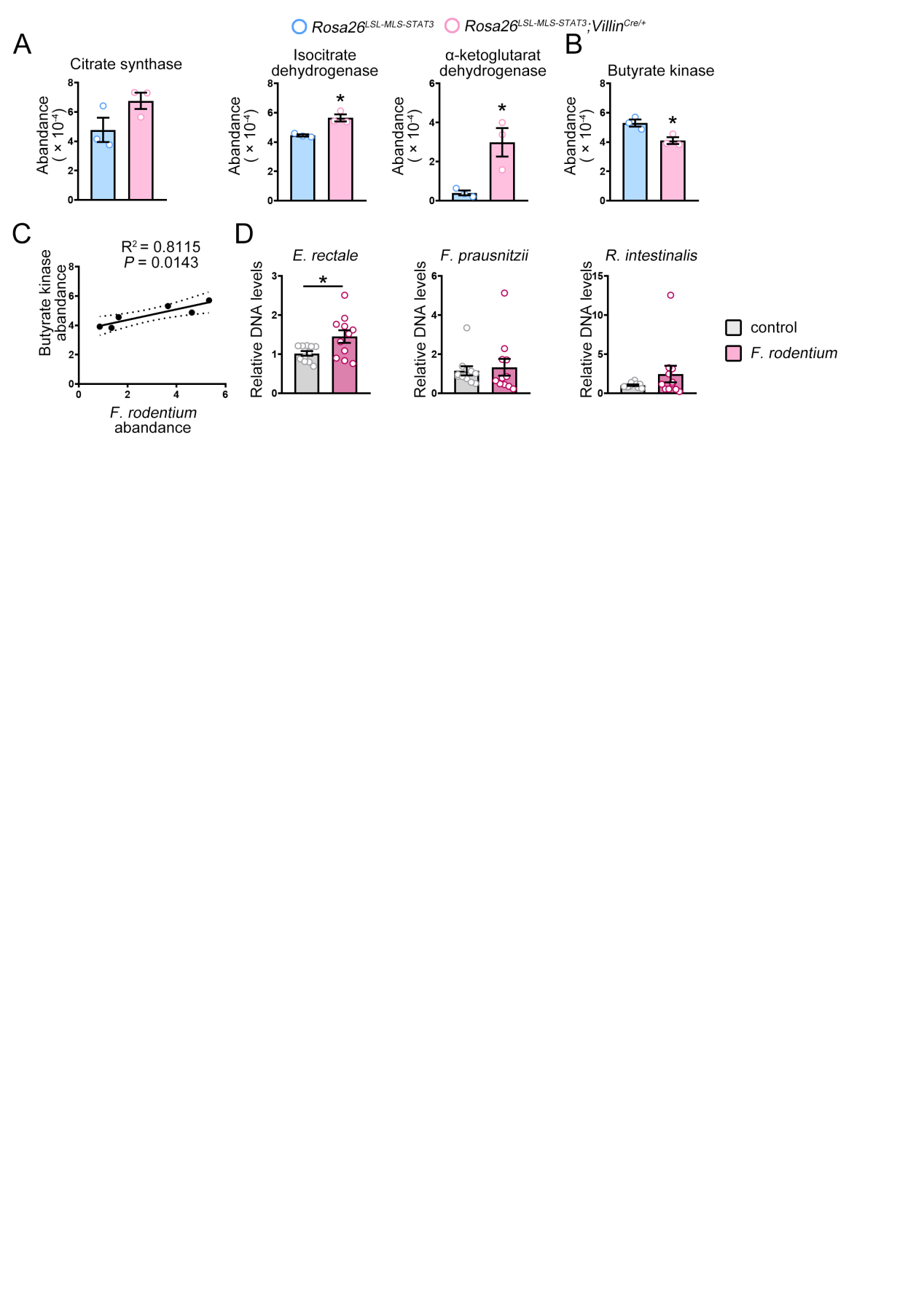
**

**Fig.S4 Butyrate was one of the key products of *F. rodentium.* (A*-*B)** The indicated enzyme levels in feces from *Rosa^26LSL-MLS-mSTAT3^* mice and *Rosa^26LSL-MLS-mSTAT3^;Villin^Cre/+^* mice were predicted by metagenomic sequencing (n = 3 mice/group). **(C)** Pearson’s correlation analysis between the relative abundance of *F. rodentium* and the butyrate kinase expression was performed. **(D)** The relative abundances of indicated butyrate-producing probiotics were examined by real-time PCR (n = 11 mice/group). The figures show the combination of two experiments. *E. rectale, Eubacterium rectale*; *F. prausnitzii, Faecalibacterium prausnitzii*; *R. intestinalis, Roseburia intestinalis.* **P*<0.05.

**
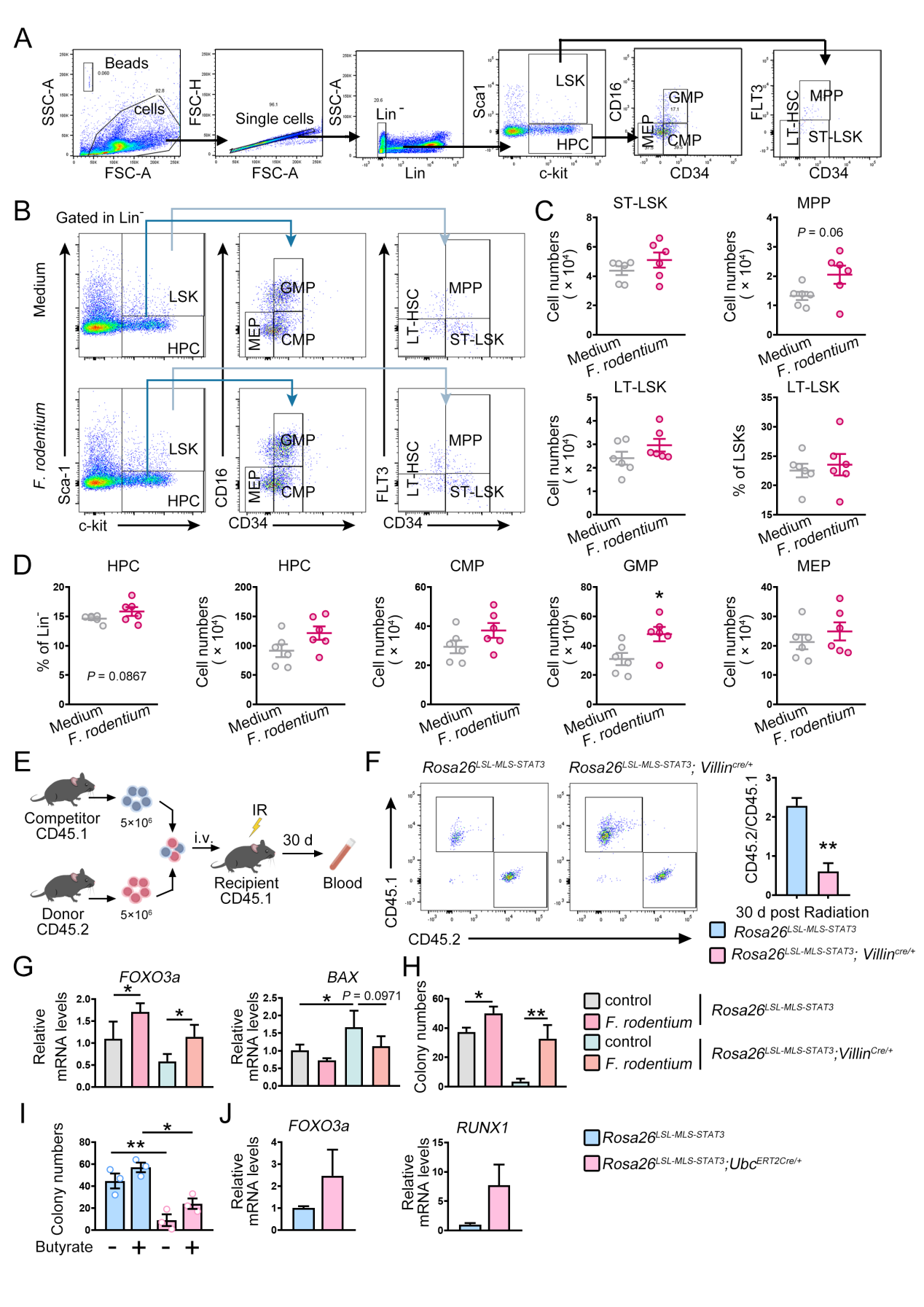
**

**Fig.S5 *F. rodentium*/butyrate promoted hematopoietic recovery. (A)** Flow cytometric sorting strategy was presented. **(B-C)** The irradiated mice were treated with culture medium or *F. rodentium*. 60 days later, the indicated subpopulations in BM were analyzed by FACS (n = 6 mice/group). Data were visualized in flow cytometry plots **(B)** and quantitatively analyzed **(C-D).** HPC, hemapoietic progenitor cells; LSK, Lin^-^Sca1^+^c-Kit^+^ cells; LT-LSK, Long-term LSK; ST-LSK, short-term LSK; GMP, granulocyte-macrophage progenitors; CMP, common myeloid progenitors; MEP, megakaryocyte-erythrocyte progenitors; MPP, multipotent progenitors. **(E-F)** Donor proportion levels were tracked by flow cytometry in recipient mice 30 days post-transplantation (n = 3 mice/group). **(B-F)** The figures shown represent a single experiment. **(G)** The relative mRNA levels of *FOXO3a* and *BAX* were assessed by real-time RT-PCR (n = 3 mice/group). **(H-I)** Colony formation assays were performed, and colony numbers were counted. **(J)** Relative mRNA levels of *FOXO3a* and *RUNX1* were assessed by real-time RT-PCR. **(G-J)** The figures shown represent one of two independent experiments. **P*<0.05, ***P*<0.01.


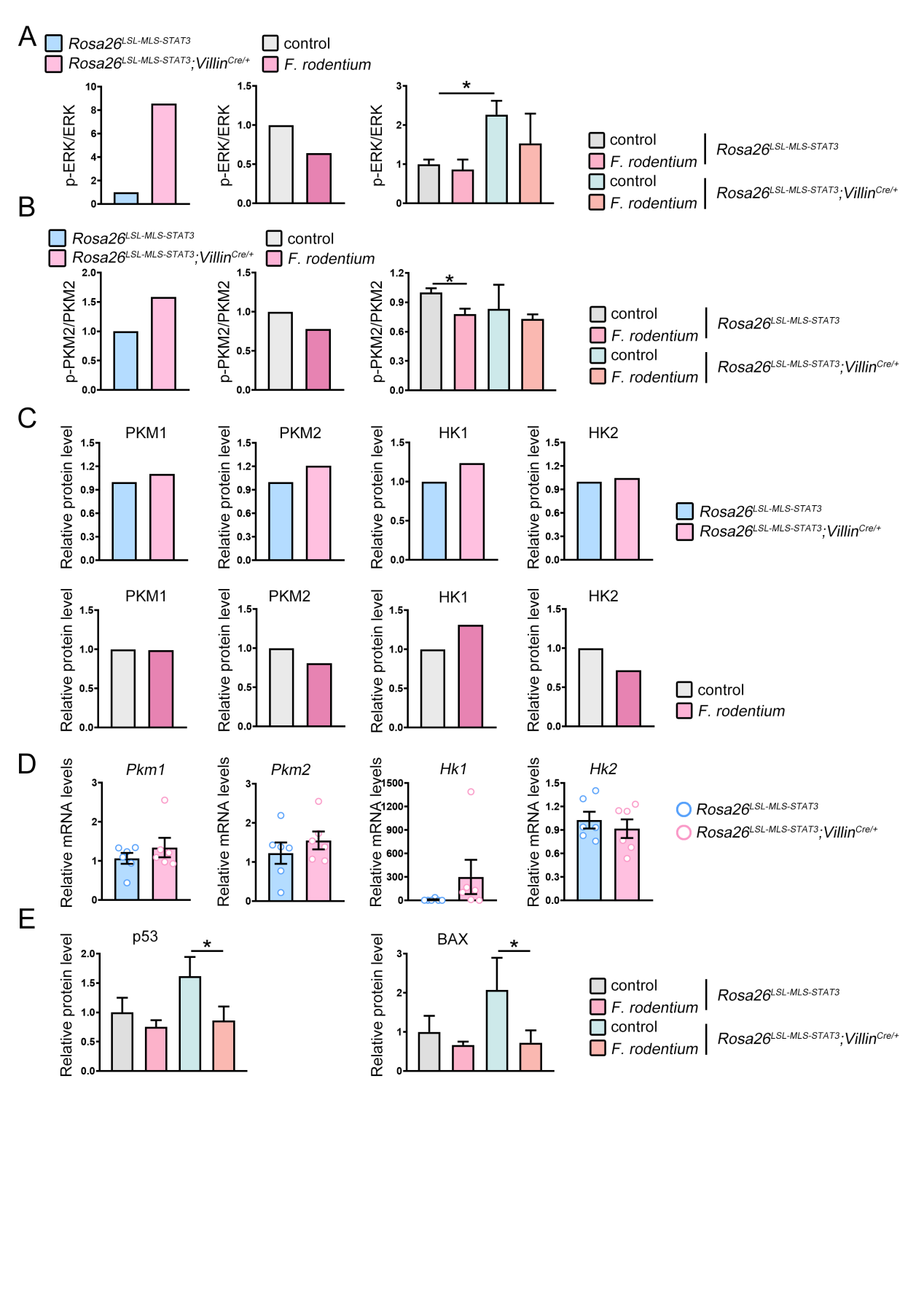


**Fig.S6 Levels of key enzymes in glycolysis were examined. (A-C)** The levels of the indicated protein were quantified by densitometry, normalized to the indicated total protein or loading control, and plotted. **(D)** The relative mRNA levels of indicated genes were examined in c-Kit^+^ cells from either IR-treated *Rosa26^LSL-MLS-mSTAT3^* mice or IR-treated *Rosa26^LSL-MLS-mSTAT3^*;*Villin^Cre/+^* mice (n = 6 mice/group). **(E)** The levels of the indicated protein were quantified by densitometry, normalized to the indicated total protein or loading control, and plotted. **(A-E)** The figures shown represent one of two independent experiments. **P*<0.05.


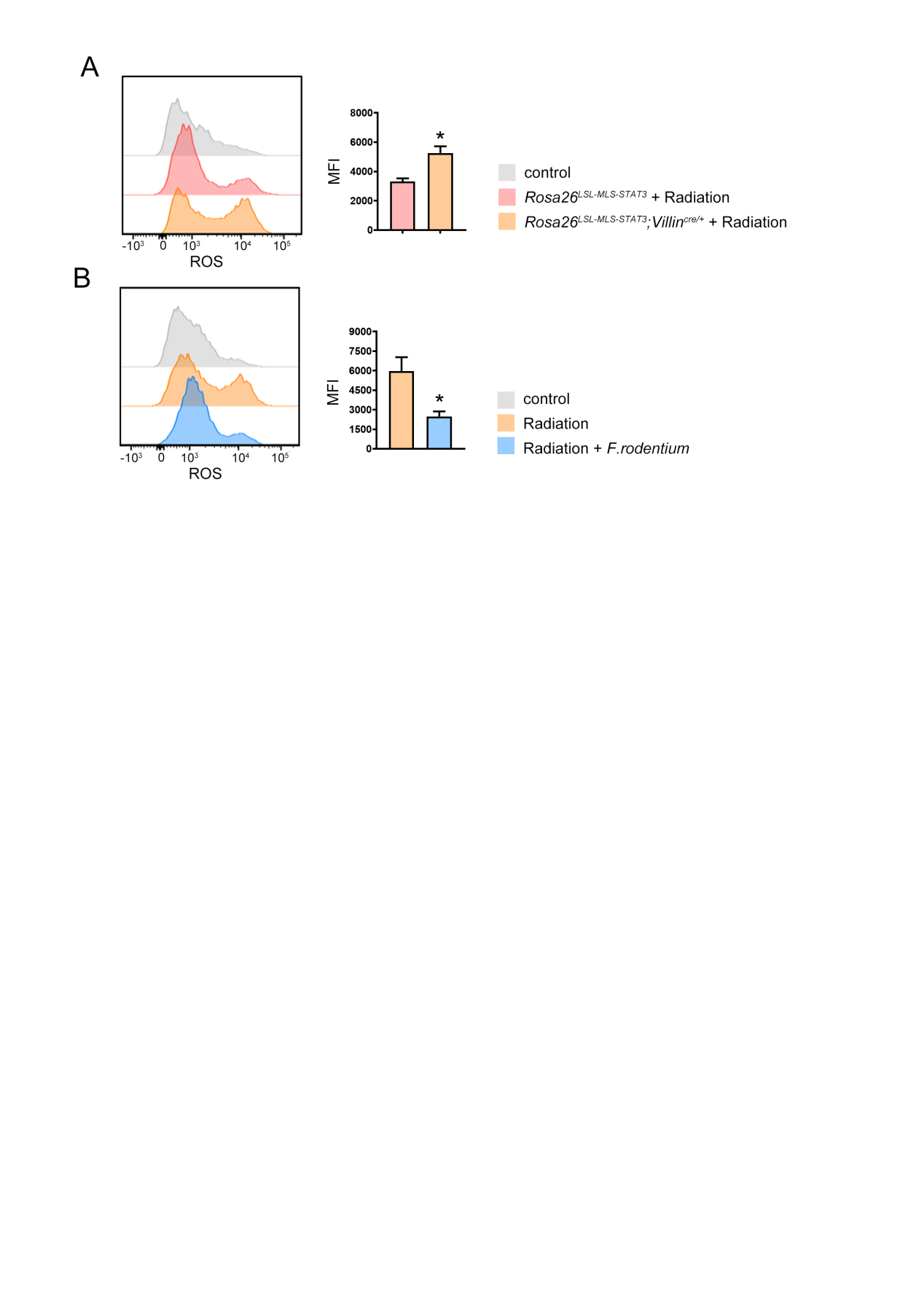


**Fig.S7 Mitochondrial STAT3 increased intracellular ROS levels. (A)** The intracellular ROS levels were assessed in IECs from irradiated *Rosa26^LSL-MLS-mSTAT3^* mice or irradiated *Rosa26^LSL-MLS-mSTAT3^*;*Villin^Cre/+^* mice (12 h post-irradiation, n = 3 mice/group). **(B)** The intracellular ROS levels were assessed in irradiated mice treated with culture medium or *F. rodentium* (12 h post-irradiation, n = 3 mice/group). **(A-B)** The figures shown represent one of two independent experiments. **P*<0.05.

**Supplementary tables**

**Table S1. The protein in mitochondria fraction.**

| Gene | Protein name | Normal water | |  | ABX | |
| --- | --- | --- | --- | --- | --- | --- |
|  |  | Peptides | Coverge(%) |  | Peptides | Coverge(%) |
| CS | Citrate synthase | 22 | 57.97 |  | 26 | 51.72 |
| IDH2 | Isocitrate dehydrogenase | 12 | 45.13 |  | 14 | 42.92 |
| LDHA | L-lactate dehydrogenase A | 9 | 31.33 |  | 14 | 37.35 |
| TIMM44 | Translocase of inner mitochondrial membrane 44 | 6 | 23.78 |  | 6 | 24.67 |
| STAT | Signal transducer and activator of transcription | 0 | 0 |  | 1 | 5.874 |

**Table S2. Clinical Score Parameters for radiation induced sickness.**

| Parameters | Score | Characterization |
| --- | --- | --- |
| A.Physical appearance | 0 | normal |
|  | 1 | lack of grooming |
|  | 2 | rough hair coat |
|  | 3 | very rough hair coat |
| B.Posture | 0 | normal |
|  | 1 | sitting in hunched position |
|  | 4 | hunched posture,head resting on floor |
|  | 6 | lying prone on cage floor/unable to maintain upright posture |
| C.Activity/  Behavior | 0 | normal |
|  | 1 | somewhat reduced/minor changes in behavior |
|  | 3 | above plus change in respiratory rate or effort |
|  | 6 | moves only when stimulated |
| D.Appetite | 0 | normal |
|  | 1 | reduced appetite |
|  | 2 | not eating since last check point |
|  | 3 | not eating for last 2 check points |
| 1. Body Weight   ((Initial weight - existing weight)/Initial weight) | 0 | normal (<5% change from initial weight) |
|  | 1 | 5-10% weight change |
|  | 2 | 10-14.9% weight change |
|  | 3 | 15-19.9% weight change |
|  | 4 | 20-24.9% weight change |
|  | 6 | > 25% weight change |

Endpoint for euthanasia: any single parameter of 6 or combined score for parameters A to E => 12.

**Table S3. Fecal DNA PCR primers in this study.**

| Gene name | Direction | Sequence |
| --- | --- | --- |
| *Parabacteroides_merdae* | Forward  Reverse | 5’-AGTGTGTTTGAGGTAGGCGG-3’  5’-ACGCTTTCGCTGTAGAGCTT-3’ |
| *Lactobacillus_johnsonii* | Forward  Reverse | 5’-GTGCAAAGCCGGATGAATGT-3’  5’-GGCTTTTCAAGAAGTGGCGT-3’ |
| *Akkermansia_muciniphila* | Forward  Reverse | 5’-CAGCACGTGAAGGTGGGGAC-3’  5’-CCTTGCGGTTGGCTTCAGAT-3’ |
| *Faecalibaculum_rodentium* | Forward  Reverse | 5’-CCGGGAATACGCTCTGGAAA-3’  5’-GCCAACCAACTAATGCACCG-3’ |
| *Bifidobacterium_pseudolongum*  *Faecalibacterium_prausnitzii*  *Eubacterium_rectale*  *Roseburia_intestinalis*  *Holdemanella_biformis*  *Roseburia .spp* | Forward  Reverse  Forward  Reverse  Forward  Reverse  Forward  Reverse  Forward  Reverse  Forward  Reverse | 5’-CCCTTTTTCCGGGTCCTGT-3’  5’-ATCCGAACTGAGACCGGTT-3’  5’-TACTGCTTGGCGTCCTTCTC-3’  5’-CGATTGCCACGTCCTGACTG-3’  5’-GCAGGAAGCACCACTTTCAC-3’  5’-TGCCTGACCAATCGGATTTCC-3’  5’-ACTCCTACGGGAGGCAGCAG-3’  5’-ATTACCGCGGCTGCTGG-3’  5’-GCTAAGGCCATGAACATGGA-3’  5’-GCCGTCCTCTTCTGTTCTC-3’  5’-TACTGCATTGGAAACTGTCG-3’  5’-CGGCACCGAAGAGCAAT-3’ |
| *Butyricimonas.spp* | Forward  Reverse | 5’-GGTGAGTAACACGTGTGCAAC-3’  5’-TACCCCGCCAACTACCTAATG-3’ |
| *Universal 16S rDNA* | Forward  Reverse | 5’-ACTCCTACGGGAGGCAGCAG-3’  5’-ATTACCGCGGCTGCTGG-3’ |

**Table S4. Real time RT-PCR primers in this study.**

| Gene name | Direction | Sequence |
| --- | --- | --- |
| *BAX* | Forward  Reverse | 5’-ATGATTGCTGACGTGGAC-3’  5’-CTAGCAAAGTAGAAGAGGGC-3’ |
| *Puma* | Forward  Reverse | 5’-CAAGAAGAGCAGCATCGACA-3’  5’-TAGTTGGGCTCCATTTCTGG-3’ |
| *FOXO3a* | Forward  Reverse | 5’-CCTATGCCGACCTGATCACC-3’  5’-ATTCTGAACGCGCATGAAGC-3’ |
| *Pkm1* | Forward  Reverse | 5’-GCTGTTTGAAGAGCTTGTGC-3'  5’-TTATAAGAGGCCTCCACGCT-3' |
| *Pkm2* | Forward  Reverse | 5’-GTCTGGAGAAACAGCCAAGG-3'  5’-CGGAGTTCCTCGAATAGCTG-3' |
| *Hk1* | Forward  Reverse | 5’-CCAAAATAGACGAGGCCGTA-3’  5’-TTCAGCAGCTTGACCACATC-3’ |
| *Hk2*  *RUNX1* | Forward  Reverse  Forward  Reverse | 5’-GAAGATGATCAGCGGGATGT-3’  5’-GCCAGTGGTAAGGAGCTCTG-3'  5’-CGAAGACATCGGCAGAAACT-3’  5’-GCTGAGGGTTAAAGGCAGTG-3’ |
| *Actb* | Forward  Reverse | 5’-GTGGGAATGGGTCAGAAGGA-3’  5’-CTTCTCCATGTCGTCCCAGT-3’ |
